# Mean Field Games and Ideal Free Distribution

**DOI:** 10.1007/s00285-025-02276-z

**Published:** 2025-09-18

**Authors:** Robert Stephen Cantrell, Chris Cosner, King-Yeung Lam, Idriss Mazari-Fouquer

**Affiliations:** 1 Department of Mathematics, University of Miami, 1365 Memorial Drive, Coral Gables, FL 33146 USA; 2 Department of Mathematics, The Ohio State University, 100 Math Tower, 231 W 18th Ave., Columbus, OH 43210 USA; 3 CEREMADE, Paris Dauphiné Université, Place du Maréchal de Lattre de Tassigny, 75775 PARIS, Cedex 16 France

**Keywords:** Mean field game, Ideal free distribution, Reaction-diffusion equations, Hamilton-jacobi, Bellman equation, Long-time average, 35Q89, 91A16, 35Q92, 92D25

## Abstract

The ideal free distribution in ecology was introduced by Fretwell and Lucas to model the habitat selection of animal populations. In this paper, we revisit the concept via a mean field game system with local coupling, which models a dynamic version of the habitat selection game in ecology. We establish the existence of classical solution of the ergodic mean field game system, including the case of heterogeneous diffusion when the underlying domain is one-dimensional and further show that the population density of agents converges to the ideal free distribution of the underlying habitat selection game, as the cost of control tends to zero. Our analysis provides a derivation of ideal free distribution in a dynamical context.

## Introduction

How organisms move to select their habitat is a central question in ecology. From an evolutionary perspective, organisms tend to adopt strategies in order to optimize their fitness (Haugen et al. 2006). The optimization viewpoint is applied extensively in the study of how adaptation can occur in animal foraging behavior (Bartumeus et al. 2031; Watanabe et al. 2014), in habitat choices (Křivan 1996), as well as in the migration process of many organisms (Alerstam 2011; Yoshioka et al. 2019). In most cases, it is far from a straightforward optimization problem of an individual navigating in a static environment, given that environmental productivity and habitat suitability depend on interactions between individuals inhabiting a given location. Hence, the game theoretical framework (Smith and Price 1973) is widely applied, leading to the search for Nash equilibria of a game with many players in which each player can anticipate the average response of others, so as to adopt a best response strategy.

### The Ideal Free Distribution

An important paradigm was presented by Fretwell and Lucas (1969); Fretwell (1972), who introduced the ideal free distribution (IFD), which can be understood as a Nash equilibrium concept in a habitat selection game (see Lemma 3.2). Their simplest model predicts that as all individuals move around freely until they cannot do any better in terms of obtaining resources, the local fitness of individuals will be equal in all occupied habitats, whereas the local fitness in the unoccupied habitat is less than or equal to the occupied ones (Holt and Barfield 2001). Later, Cressman and Křivan (2006); Křivan et al. (2008) proved that the patch selection strategy producing an IFD is an example of an evolutionarily stable strategy (ESS), *i.e.* a strategy which is stable with respect to any other patch selection strategy.

More precisely, consider a smooth bounded spatial domain $\Omega$ and let *F*(*x*, *m*) be the local fitness at location *x*, given the local population density *m*, and assume that *F*(*x*, *m*) decreases in *m*. The theory of ideal free distribution predicts the following:

(I) Suppose the spatial location *x* is occupied while the location *y* is unoccupied, i.e. $m(x) >0$ while $m(y) = 0$. Then we must have $F(x,m(x)) \ge F(y,m(y))$, for individuals at location *x* would otherwise leave and move to location *y*;
(II) Suppose the spatial location *x* and *y* are occupied. Then local density must be adjusted so that $F(x,m(x)) = F(y,m(y))$ for $x,y \in \{x':~ m(x')>0\}$.

In a different line of research focusing on the evolution of dispersal, Hastings (Hastings 1983) showed that lower dispersal rates are selected among phenotypes that are randomly dispersing in a spatially heterogeneous but temporally constant environment, in the sense that for two phenotypes which are identical except for their dispersal strategies, the one with lower dispersal rate always competitively ousts the one with higher dispersal rate. See (Cantrell and Lam 2021; Dockery et al. 1998; Lam and Lou 2024) for mathematical results in a more sophisticated context. A closer examination of the results reveals that random dispersal creates a mismatch between population distribution and the carrying capacity. This mismatch allows for the possible invasion by phenotypes with exotic dispersal strategies. In McPeek and Holt (1992), McPeek and Holt analyzed spatially discrete models and found that selection favors dispersal strategies that do not create such mismatches. Later on, the evolutionary stability of such strategies was proved in Cantrell et al. (2007) for spatially discrete models, and in Cantrell et al. (2010) for reaction-diffusion-advection systems. It is interesting to underline another point of Holt and Barfield (2001): many dispersal behaviors, which are not necessarily ideal or free, can lead to an ideal free distribution. Indeed, it was proved in Cantrell et al. (2007, 2010) that a class of dispersal behavior, for which the movement of the organism only depends on local (but not global) spatial information, is enough to produce IFD.

As aforementioned, the IFD can be regarded as the Nash equilibrium of a habitat selection game, and as such it does not address the mechanisms and dynamics that might lead to IFD. The analyses using the adaptive dynamics framework in Hastings (1983); Cantrell et al. (2010) partially addressed this problem by showing that dispersal strategies producing IFD are both evolutionarily stable strategies (ESS), as well as neighborhood invader strategies (NIS). Roughly speaking, a strategy is an ESS if it is an evolutionary endpoint, while it is an NIS if phenotypes with such a dispersal strategy (should they arise by random mutation) can always dominate and outcompete whichever resident strategies that were present. Precisely, consider the following reaction-diffusion-advection model

1.1 $$\begin{aligned} m_t = \textrm{div}(\mu \nabla m - \vec {P}(x)m) + m(K(x) - m) \qquad \text { for }t>0,~ x\in \Omega , \end{aligned}$$

with no-flux boundary condition on $\partial \Omega$, modeling a population density *m*(*x*, *t*) whose members move with a combination of diffusion with rate $\mu >0$ and a biased movement following the vector field $\vec {P}(x)$. Under the framework of adaptive dynamics, and by regarding the vector field $\vec {P}$ as *strategy*, it is proved that the set of evolutionarily stable strategies coincides with those strategies $\vec {P}$ whose corresponding stationary distribution ${\hat{m}}(x)$ leads to equilibration of fitness, i.e.

$$\begin{aligned} F(x,{\hat{m}}(x)) = K(x) - {\hat{m}}(x) = \text { constant}. \end{aligned}$$

Hence, it is necessary that the population distribution exactly matches the carrying capacity *K*(*x*) at an ESS (Cantrell et al. 2010). (In principle we could consider other general logistic-type growth rate where $s\mapsto F(x,s)$ is strictly decreasing.) This is also true when the environmental conditions vary periodically as well, under mild conditions (Cantrell et al. 2021).

Besides the framework of adaptive dynamics, another explicit type of game dynamics was introduced by Taylor and Jonker (1978). These dynamics, called the “replicator equations", are constructed to model situations in which there is an instantaneous change in the frequency of different strategies due to their differing relative fitness. The Folk Theorem of this framework relates the dynamical stability of the replicator equation with the Nash equilibrium property or evolutionary stability of a given strategy. A wider class of dispersal dynamics, including best response dispersal strategies, was considered by Křivan et al. (2008). Recently, Ambrosio et al. (2021) introduced and proved the well-posedness of spatially heterogeneous replicator models in continuous space and derived the limit as the number of agents tends to infinity.

### Our main objective

In this paper, we interpret the framework of mean field games (MFG) in terms of evolution of dispersal, in which individual movement is governed by a controlled diffusion process so as to optimize an objective functional. The objective functional incorporates the effect of the cost of control, the payoff function *F*(*t*, *x*) which is perturbed by a mean field distribution *m*(*t*, *x*) of the conspecifics. For each fixed cost of control, we will first develop the existence and uniqueness of solution of MFG, which corresponds to the Nash equilibrium of the mean field game. Next, we will show that as the cost of control/dispersal goes to zero, the overall population distribution of individuals converges to the IFD. This provides a dynamical optimization through which IFD can be achieved. For the precise statement of our main results, see Theorems 3.4 and 3.5.

### Mean Field Games

The notion of MFG was considered in the economics literature by Jovanovic and Rosenthal (1988), in the engineering literature by Huang et al. (2006), and independently and around the same time by the mathematicians Lasry and Lions (2007). MFG models are a set of PDEs used to approximate an infinite number of players behaving as a Nash equilibrium with respect to a differential game. In the game, each individual has knowledge of its own space-time coordinate, and the empirical distribution of the other players. In contrast to existing adaptive dynamics models where one studies the invasion of trait/phenotype by allowing (usually two) populations with prescribed dispersal strategies to compete (Hastings 1983; Dockery et al. 1998; Cantrell et al. 2010), MFG focuses on the selection at the level of an individual, which is able to optimize its performance as measured by a suitable payoff functional *K*(*x*) which is perturbed by the mean field term *m*(*t*, *x*) representing the average behavior of the infinite number of agents. Of course, the complexity of the differential game becomes intractable as the number of individuals becomes large. Thus, MFG considers the special solutions in which all the players are identical, meaning they are governed by an identical (albeit independent) controlled diffusion process and are optimizing a symmetric objective. In other words, MFG models symmetric Nash equilibria, where the average player chooses a behavioral strategy which is optimal given mean field terms where all other agents also adopt the given strategy. Roughly speaking, a typical individual in the MFG solution uses both the information on (i) the net payoff function *K*(*x*) and (ii) how the mean field distribution *m*(*t*, *x*) depends on the choice of individual feedback control $\alpha (t,x)$, to adopt a control that is optimal (balancing the cost of control with the perturbed payoff function $F(t,x)=K(x) - m(t,x)$) in anticipation of how the entire population is expected to distribute in the future up to the terminal time. The questions of existence, uniqueness and qualitative properties of this equilibrium are at the core of the mathematical difficulties of MFG.

MFG has a number of applications in economic theory (Carmona 2020; Guéant 2009; Jovanovic and Rosenthal 1988), cryptocurrency and Bitcoin mining (Bertucci et al. 2024) and financial engineering (Carmona et al. 2010). It is also applied to model biological phenomena such as animal swarming (Morale et al. 2005; Stella et al. 2021) and diel migration in phytoplankton (Mazuryn and Thygesen 2023). For the latter area, Thygesen and his coauthors developed a different approach to the connection between the ideal free distribution and mean field games in Frølich and Thygesen (2022) and applied it to predator-prey systems. Its mathematical formulation is more explicitly game theoretical than ours, in the spirit of Křivan et al. (2008), and does not use the formulation of Lasry and Lions (2007) directly. The models in Frølich and Thygesen (2022) assume that movement takes place on a fast timescale so that it instantaneously reaches an equilibrium in space, as opposed to our starting assumption of diffusive movement. The analysis in Frølich and Thygesen (2022) is based on variational inequalities. Related ideas are developed in Frølich and Thygesen (2022). In Thygesen and Mazuryn (2022) the authors formulate a mean field game for the diel migration of copepods. They use an explicit formula for cost of motion and note that any equilibrium in their model would correspond to an ideal free distribution. In a later paper (Mazuryn and Thygesen 2023) they extend those ideas and derive a system to characterize their mean field game which is similar to the one we will consider analytically in this paper.

## Model Formulation

Within the MFG framework we consider a population of individual agents who can assess the quality of their surrounding environments, the spatial distribution of conspecifics, and are able to move freely.

Mathematically, assume that a representative agent is governed by the following controlled stochastic differential equation (SDE):

2.1 $$\begin{aligned} dX_t = \alpha (t,X_t) dt + \sqrt{{2\mu (X_t)}} dB_t, \quad X_0 = x \in \Omega \subseteq \mathbb {R}^d, \end{aligned}$$

where *x* is the initial state/location, $\alpha _t = \alpha (t,X_t)$ represents the feedback control terms and $B_t$ is a standard Brownian motion with a state-dependent coefficient $\mu (x)$ which is smooth and bounded from above and below by positive constants. More precisely, the above SDE applies when $X_t$ is in the interior of $\Omega$, while on the boundary $\partial \Omega$ it is reflected as modeled by a Skorokhod problem (Lions and Sznitman 1984), leading to the no-flux boundary condition (2.3). Consistent with the notion of symmetric Nash equilibrium, we assume that all agents are indistinguishable and follow the above SDE with independent noise. Let a finite time horizon $T>0$ be fixed; if every agent is independent and is governed by the same diffusive law given above, the population density $m^T(t,x)$ of agents is given by the forward Fokker-Planck equation (Pavliotis 2014):

2.2 $$\begin{aligned} \partial _t m = \Delta ( \mu m) - \textrm{div}( \alpha m) \quad \text { in }\Omega \times (0,T), \quad m(0,x)=m_0(x)\quad \text { in }\Omega . \end{aligned}$$

with boundary condition (thanks to the Skorokhod formulation)

2.3 $$\begin{aligned} \nu \cdot (\mu \nabla m^T - m^T \alpha )=0 \quad \text { on }\partial \Omega \times (0,T), \end{aligned}$$

where $\nu$ is the unit outward normal vector on $\partial \Omega$. The representative agent then seeks to optimize a payoff functional $\mathcal {J}^T(t,x;\alpha )$ over a finite time horizon [0, *T*], i.e.

2.4 $$\begin{aligned} u^T(t,x) = \inf _{\alpha } \mathcal {J}^T(t,x,;\alpha ) \end{aligned}$$

where the payoff functional $\mathcal {J}^T(t,x,;\alpha )$ depends on the behavior of the population density $m^T$ of all players, in addition to other factors:

2.5 $$\begin{aligned} \mathcal {J}^T(t,x;\alpha )=E_{t,x} \left\{ \int _t^T \frac{\epsilon }{2} |\alpha _s|^2 - \frac{1}{\epsilon }F(X_s,m^T(s,X_s))\,ds + G(X_T)\right\} . \end{aligned}$$

Here we take a quadratic cost of control for simplicity, as it represents the square of velocity, which represents the energy cost to implement the control (see further discussion in Section 4.1), and $\alpha =\{\alpha _s\}_s$ is a nonanticipative control process, i.e. for each *s*, $\alpha _s$ can depend on knowledge of the process $\{X_{s'}\}_{0 \le s' \le s}$ up to time *s*. Next, we discuss the choice of our scaling factor $\epsilon$ in (2.5). First, note that the optimal control $\alpha$ (that minimizes $\mathcal {J}^T$) will not change even if we multiply $\mathcal {J}^T$ by any function $h(\epsilon )$. Hence $\epsilon$ is genuinely the ratio between the control cost running cost. The specific choice of the coefficients $\tfrac{\epsilon }{2}$ and $\tfrac{1}{\epsilon }$ is to prevent the value function from converging to zero or infinity as $\epsilon \rightarrow 0$.

To derive the IFD, we will first let $T\rightarrow \infty$ to connect with the ergodic MFG and then consider the limit $\epsilon \rightarrow 0$ when the cost of control becomes negligible. Further discussion can be found in Section 4.

A typical choice of *F*(*x*, *m*) is given in logistic form:

2.6 $$\begin{aligned} F(x,m) =r(x)\left( 1- \frac{m}{K(x)}\right) \end{aligned}$$

where *r*(*x*) and *K*(*x*) are the intrinsic growth rate and the carrying capacity, respectively. When *F* is independent of *m*, (2.4) becomes a typical stochastic optimal control problem, and has been applied extensively in mathematical biology, such as in bird migration (Alerstam 2011). Motivated by the differential games of many players, the MFG formulation incorporates the consideration that each player is *playing the field*, which means that the individual is optimizing its control strategy $$\{\alpha _t\}$$ while anticipating the density $$ m^T(t,x)$$ of other players.

By considering for the moment the density of $m^T(t,x)$ as given, and requiring that the individual behavior is consistent with the payoff $(t,x)\mapsto F(x,m^T(t,x))$, it follows from classical theory (Fleming and Soner 2006) that the optimal control is necessarily given as a feedback control proportional to the gradient of the value function *u*

2.7 $$\begin{aligned} \alpha = -\frac{1}{\epsilon }\nabla u^T(t,X_t) \end{aligned}$$

where $u^T(t,x)$ is the value function associated with this minimization (2.4), which can be characterized as the unique viscosity solution to the Hamilton-Jacobi-Bellman equation

$$\begin{aligned} 0 = \max _{\alpha } \left[ -(u^T)_t - \mu \Delta u^T - \alpha \cdot \nabla u^T - \frac{\epsilon }{2}|\alpha |^2 + \frac{1}{\epsilon }F(x, m^T(t,x)) \right] . \end{aligned}$$

The Hamilton-Jacobi-Bellman equation can be written as follows:

2.8 $$\begin{aligned} {\left\{ \begin{array}{ll} - (u^T)_t - \mu \Delta u^T + \frac{1}{2\epsilon }|\nabla u^T|^2 + \frac{1}{\epsilon }F(x,m^T(t,x))=0 & \text { for }t\in [0,T],~x\in \Omega ,\\ u^T(T,x) = G(x) & \text { for }x\in \Omega . \end{array}\right. } \end{aligned}$$

Finally, the reflecting boundary condition of the diffusion process keeps the process inside $\bar{\Omega }$, hence the value function satisfies the Neumann condition (Puterman 1977, Theorem 4.1), which says that the controller cannot lower the cost by pushing the state outside the domain:

2.9 $$\begin{aligned} \nu \cdot \nabla u^T = 0 \quad \text { for }t \in (0,T),~ x \in \partial \Omega . \end{aligned}$$

Upon substituting (2.7) into (2.2) and (2.3), then combining with (2.8)-(2.9), we obtain the finite horizon MFG model with local coupling (see Cardaliaguet and Porretta 2020):

2.10 $$\begin{aligned} {\left\{ \begin{array}{ll} \partial _t u^T = - \mu \Delta u^T + \frac{1}{2\epsilon } |\nabla u^T|^2 + \frac{1}{\epsilon }F(x,m^T(t,x)) & \text { for }t\in [0,T],~x\in \Omega ,\\ \partial _t m^T = \Delta (\mu m^T)+ \textrm{div} (m^T \frac{\nabla u^T}{\epsilon })& \text { for }t\in [0,T],~x\in \Omega ,\\ \nu \cdot \nabla u^T = 0 & \text { for }t\in [0,T],~x\in \partial \Omega ,\\ \nu \cdot (\mu \nabla m^T + m^T\frac{\nabla u^T}{\epsilon } ) = 0& \text { for }t\in [0,T],~x\in \partial \Omega ,\\ m^T(0,x) = m_0(x),\quad u^T(T,x) = G(x) & \text { for }x\in \Omega . \end{array}\right. } \end{aligned}$$

When the parameter $\epsilon >0$ (which appears originally in the cost functional in (2.5)) is small, then the cost of control becomes small and the drift due to control dominates over the standard noise due to diffusion in the Fokker-Planck equation governing the population density $m^T(t,x)$. It is this combination of large and optimal drift and a bounded diffusive movement that together enables the ideal free distribution.

### The Stationary Problem

It is natural to investigate the behavior of the MFG system (2.10) as the horizon *T* tends to infinity. In fact, it can be shown that the influence of the initial/terminal data $(m_0, G)$ vanishes as $T\rightarrow \infty$ (see Proposition 2.1 below), and that the long-time average can be approximated by the following stationary ergodic problem with unknowns $(\lambda , u(x), m(x))$. (For later purposes, we also denote the solution by $(\lambda ^\epsilon , u^\epsilon , m^\epsilon )$ to emphasize the dependence on $\epsilon$.)

2.11 $$\begin{aligned} {\left\{ \begin{array}{ll} \lambda - \epsilon \mu \Delta u + \frac{1}{2} |\nabla u |^2 +F(x, m(x))=0 & \text { for }x\in \Omega ,\\ -\epsilon \Delta (\mu m) - \textrm{div}\left( m {\nabla u}\right) =0 & \text { for }x \in \Omega ,\\ \nu \cdot \nabla u = \nu \cdot [\nabla (\mu m) + m \nabla u] = 0 & \text { for }x\in \partial \Omega ,\\ \int _\Omega u\,dx = 0 \quad \text { and }\quad \int _\Omega m\,dx = \bar{m}_0:= \int _\Omega m_0\,dx. & \end{array}\right. } \end{aligned}$$

Here $\lambda \in \mathbb {R}$ is called the ergodic constant, or optimal long-time average reward. This system is central in the study of the long-time behavior of MFG systems, and has been the topic of a systematic study when $\mu$ is constant and when *F* satisfies stronger regularity assumptions. The two main references are Cardaliaguet (2013); Cardaliaguet et al. (2012). It is important to point out that in the first-order case, that is, when ${\varepsilon }=0$, the existence of solutions to the ergodic system under several assumptions is linked to the weak KAM theory; we refer to Cardaliaguet (2013) for a discussion of this aspect of the theory. In Appendix B, we provide some existence results for the stationary problem. In case $\mu$ is a constant, the existence of the solution is due to Cardaliaguet et al. (2012). In this paper, we also derive the existence of classical solutions when $\mu$ is nonconstant under the limitation that $\Omega$ is one-dimensional.

The ergodic system (2.11) can be interpreted as follows: each agent seeks to minimize his/her ergodic cost ${u}(x) = \inf _\alpha \mathcal {J}(x,\alpha )$, where $\mathcal {J}$ is the ergodic cost function

$$\begin{aligned} {u}(x) = \inf _\alpha \mathcal {J}(x,\alpha ) = \inf _\alpha \limsup _{T\rightarrow \infty } \mathbb {E} \left[ \frac{1}{T}\int _0^T \frac{\epsilon }{2}|\alpha (X_t)|^2 - \frac{1}{\epsilon }F(X_t, m^{(\alpha )}(X_t))\,dt \right] , \end{aligned}$$

where $\alpha = \alpha (x)$ is a feedback control, and $X_t$ is the solution to the SDE

$$\begin{aligned} dX_t = \alpha (X_t) dt + \sqrt{2\mu (X_t)}dW_t \quad \text { such that }\quad X_0 = x, \end{aligned}$$

and ${m}^{(\alpha )}(x)$ is the stationary population density which satisfies

2.12 $$\begin{aligned} {\left\{ \begin{array}{ll} - \Delta (\mu m) + \textrm{div}(\alpha m ) = 0 \quad \text { in }\Omega ,\\ \nu \cdot [ \nabla (\mu m) + \alpha m]=0 \quad \text { on }\partial \Omega , \\ \int _\Omega m \,dx = {\bar{m}}_0. \end{array}\right. } \end{aligned}$$

Here, we state a convergence result of the solution of (2.10) to that of the ergodic problem (2.11). For convenience, we provide a version here and refer the reader to ( Cardaliaguet et al. (2012), Theorems 2.1 and 3.1) and (Cardaliaguet and Porretta 2020, Theorem 1.14) for more precise results. We need the monotonicity condition

1. $$F(x,s)-F(x,s') \ge 0$$ for $$x \in \Omega ,~\text {and}~s'\ge s.$$
2. $F \in \textrm{Lip}_{loc}([0,\infty )\times \bar{\Omega })$ and there is $c_1>0$ such that $F(x,s)-F(x,s') \ge c_1 (s'-s)$ for $x \in \Omega ,~\text {and}~s'\ge s.$

#### Proposition 2.1

Let $(\lambda , u(x), m(x)) \in \mathbb {R}\times C^{2+\beta }(\bar{\Omega }) \times C^{2+\beta }(\bar{\Omega })$ be a classical solution of the ergodic problem (2.11), and for each $T>0$, let $(u^T(t,x),m^T(t,x))$ be a solution of (2.10) such that

$$\begin{aligned} m_0, G \in C^2(\bar{\Omega }) \quad \text { and }\quad \inf _\Omega m_0 >0. \end{aligned}$$

Define $$\theta ^T, \nu ^T:[0,1]\times \Omega \rightarrow \mathbb {R}$$ by

2.13 $$\begin{aligned} \theta ^T(s,x) = u^T(sT,x) \quad \text { and }\quad \nu ^T(s,x) = m^T(sT,x). \end{aligned}$$

- Suppose (F1) holds. Then
  2.14 $$\begin{aligned} \iint _{(0,1)\times \Omega } (\nu ^T + m)|\nabla \theta ^T - \nabla u|^2\,dxdt \le \frac{C}{T} \end{aligned}$$
  2.15 $$\begin{aligned} \left| \iint _{(0,1)\times \Omega } (-F(x, \nu ^T) + F(x, m))(\nu ^T - m)\,dxdt\right| \le \frac{C}{T} \quad \text { for }T \ge 1. \end{aligned}$$
- Suppose (F2) holds. Then
  2.16 $$\begin{aligned} \Vert \nu ^T- m\Vert _{L^2((0;1)\times {\Omega })}+\Vert {{\nabla }} \theta ^T-{{\nabla }} u\Vert _{L^2((0;1)\times {\Omega })}\underset{T\rightarrow \infty }{\rightarrow }0. \end{aligned}$$
  2.17

(See Appendix A for the proof.)

In a certain sense, if individuals behave optimally, then we expect the overall population to organize into a stationary distribution, i.e. $m^T(t,x) \approx m(x)$ over a sufficiently long time horizon. In the next section, we will characterize the population distribution *m*(*x*) when the cost $\epsilon$ of control is small and connect it with the concept of the ideal free distribution.

## Deriving the ideal free distribution

It is sometimes mathematically more convenient to work with the following definition of IFD as a variational inequality (Kinderlehrer and Stampacchia 2000), which implies (I) and (II) in the introduction (see Lemma 3.2 below).

### Definition 3.1

We say that a nonnegative function ${\hat{m}} \in C(\bar{\Omega })$ is an IFD if

3.1 $$\begin{aligned} \int _\Omega F(x,{\hat{m}}(x)) m(x)\,dx \le \int _\Omega F(x,{\hat{m}}(x)){\hat{m}}(x)\,dx \end{aligned}$$

for any $0\le m \in L^1(\Omega )$ such that $\int _\Omega m \,dx = \int _\Omega {\hat{m}}\,dx$.

Here $C(\bar{\Omega })$ (resp $L^1(\Omega )$) denotes the class of functions which are continuous on $\bar{\Omega }$ (resp. integrable on $\Omega$).

The above characterization by a variational inequality is consistent with the notion of IFD being the result of selection at the individual level. Indeed, suppose $\hat{m}(x)$ is IFD, then one can regard the vast majority of individuals playing the mixed strategy $\hat{m}(x)$ implying that the fitness of a *typical* individual is given by the right-hand side of (3.1), which is necessarily greater than or equal to the fitness of an individual with an arbitrary mixed strategy *m*(*x*), as given by the left-hand side of (3.1).

Also, Definition 3.1 is consistent with the general statement of what is meant by an IFD, as outlined in (I) and (II) in the introduction, as is shown below.

### Lemma 3.2

Suppose ${\hat{m}} \in C(\bar{\Omega })$. Then ${\hat{m}}$ is an IFD according to Definition 3.1 if and only if then there exists a constant $c_0 \in \mathbb {R}$ such that

  (i) $F(x,{\hat{m}}(x)) \equiv c_0$ is constant in $\textrm{supp}\,{\hat{m}}$, and
  (ii) $F(x,{\hat{m}}(x)) \le c_0$ for all $x \in \Omega$.

### Proof

Without loss of generality, suppose $\int _\Omega \hat{m}\,dx=1$. The “$\Leftarrow$” part of the assertion is obvious, so we prove the “$\Rightarrow$" part below. Define

$$\begin{aligned}c_0 = \int _\Omega F(x, {\hat{m}}(x)){\hat{m}}(x)\,dx.\end{aligned}$$

By letting a sequence of $m=m_j \rightarrow \delta _{x'}$ (the Dirac mass supported at $x'$), we deduce from (3.1) that

3.2 $$\begin{aligned} F(x',{\hat{m}}(x')) \le c_0 \quad \text { for each }x' \in \Omega . \end{aligned}$$

Next, multiply (3.2) by ${\hat{m}}(x')$ and integrate over $\Omega$, we deduce that

$$\begin{aligned} c_0 = \int _\Omega F(x',{\hat{m}}(x')) {\hat{m}}(x')\,dx' \le \int _\Omega c_0 {\hat{m}}(x')\,dx' = c_0. \end{aligned}$$

Hence $F(x',{\hat{m}}(x')) = c_0$ for all $x' \in \textrm{supp}\,{\hat{m}}$. $\square$

To keep the ideas clear, we assume hereafter the special case when *F* is given by

3.3 $$\begin{aligned} F(x,s) = K(x) - s, \end{aligned}$$

for some strictly positive function $K \in C(\bar{\Omega })$.^1^

### Remark 3.3

If *F* is given by (3.3), then ${\hat{m}}(x)$ is an IFD if and only if

$$\begin{aligned} \hat{m}(x) = \max \{K(x) - \bar{\lambda },0\} \quad \text { for some }\bar{\lambda }\in \mathbb {R}, \end{aligned}$$

which is as described by Fretwell and Lucas (1969). To see that, define $\bar{\lambda }= \int F(x,{\bar{m}}){\bar{m}} \,dx$, then by Lemma 3.2(i), $\bar{m} = K - \bar{\lambda }$ in the support of ${\bar{m}}$, and that $\{{\bar{m}} \equiv 0\} \subset \{K \le \bar{\lambda }\}$.

Let $(\lambda ^\epsilon , u^\epsilon (x), m^\epsilon (x))$ be a solution to the stationary system (2.11). Then $m^\epsilon (x)$ represents the spatial population distribution as each individual behaves optimally given the information (consisting of the carrying capacity *K*(*x*), and the distribution of all players $m^\epsilon (x)$) and given the cost of control $\epsilon >0$. Furthermore, by Proposition 2.1, in any finite horizon MFG with $T\gg 1$, the population distribution of individuals is approximately equal to $m^\epsilon (x)$ a.e. in [0, *T*].

To derive the IFD, we consider the asymptotic limit when the cost of control tends to zero, i.e. $\epsilon \rightarrow 0$. We begin with a general result that holds in all dimensions, but only provides a weak convergence of $(m^{\varepsilon })_{{\varepsilon }>0}$ in the sense of measures.

### Theorem 3.4

For any ${\varepsilon }>0$ let $(\lambda ^\epsilon , u^\epsilon (x), m^\epsilon (x)) \in \mathbb {R}\times C^{2+\beta }(\bar{\Omega }) \times C^{2+\beta }(\bar{\Omega })$ be a classical solution of (2.11). Then there exists $\bar{\lambda }\in \mathbb {R}$ such that

3.4 $$\begin{aligned} \lambda ^{\varepsilon }\rightarrow \overline{\lambda }\quad \text { and } \quad m^{\varepsilon }\underset{L^2}{\rightharpoonup }\max \{K(x)-\overline{\lambda },0\}\quad \text { as }\epsilon \rightarrow 0, \end{aligned}$$

which is the IFD accoring to Definition 3.1 above. Particularly, $\overline{\lambda }$ is determined by

3.5 $$\begin{aligned} \quad \int _\Omega \max \{{K}(x) - \bar{\lambda },0\} \,dx = \bar{m}_0, \end{aligned}$$

and ${\bar{m}}_0$ is as given in (2.11).

In the next theorem, we will show the uniform convergence of $(m^{\varepsilon })_{{\varepsilon }>0}$, when $\mu$ is constant or when $\Omega$ is one-dimensional.

### Theorem 3.5

Let $(\lambda ^\epsilon , u^\epsilon (x), m^\epsilon (x)) \in \mathbb {R}\times C^{2+\beta }(\bar{\Omega }) \times C^{2+\beta }(\bar{\Omega })$ be a classical solution of the ergodic problem (2.11). Suppose that one of the following conditions holds.

- $\Omega$ is a smooth bounded domain in $\mathbb {R}^d$ for some $d >1$ and $\mu (x)$ is constant in *x*.
- $$\Omega = (0,1)$$;

Then as $$\epsilon \rightarrow 0$$,

3.6 $$\begin{aligned} \lambda ^\epsilon \rightarrow \bar{\lambda }\quad \text { and }\quad m^\epsilon (x) \rightarrow \max \{{K}(x) - \bar{\lambda },0\} \quad \text { uniformly in }\Omega . \end{aligned}$$

Here $\bar{\lambda }$ is uniquely characterized by (3.5). In particular, ${K}(x) - {m^\epsilon }(x)$ tends to a constant in the support of $\lim {m^\epsilon }$.

Recall that $(\lambda ^\epsilon , u^\epsilon , m^\epsilon )$ represents a symmetric Nash equilibrium of the differential game in which the individual payoff contains a mean field term $m^\epsilon$. The above result says that as the cost of the control tends to zero, then the symmetric Nash equilibrium of the ergodic problem approaches the IFD (see Remark 3.3). Particularly, there exists a constant $c_0$ such that for each $x \in \Omega$, one of the following holds:

- $$m^\epsilon (x) \rightarrow 0$$ as $$\epsilon \rightarrow 0$$;
- $$F(x, m^\epsilon (x)) \rightarrow c_0$$ as $$\epsilon \rightarrow 0$$.

i.e. the fitness function $F(x,m^\epsilon (x))$ becomes approximately constant in the support of the population. Furthermore, a corresponding statement holds for the finite horizon problem as well, thanks to Proposition 2.1. This gives an alternative derivation of the IFD via the framework of MFG in the stationary setting.

More generally, IFD can be observed in the MFG in the finite time horizon [0, *T*] with large enough $T>0$ as well. Indeed, it follows from Proposition 2.1 that the population $m^T(t,x) \approx m^\epsilon (x)$ for a.e. $t \in [0,T]$, except possibly near the initial and terminal times, when the initial distribution $m_0$ and the terminal payoff *G*(*x*) take effect.

Before we prove these theorems in the next section, let us establish the following a priori estimate.

### Lemma 3.6

Let $(\lambda ^\epsilon , u^\epsilon , m^\epsilon ) \in \mathbb {R} \times C^{2+\beta }(\bar{\Omega }) \times C^{2+\beta }(\bar{\Omega })$ be a solution of (2.11), then

3.7 $$\begin{aligned} |\lambda ^\epsilon | + \int _\Omega |\nabla u^\epsilon |^2\,dx + \int _\Omega |m^\epsilon |^2\,dx \le 3 \nonumber \\ \Vert K\Vert _{L^\infty } + 2\frac{m_0 \sup \limits _\Omega \mu }{\inf \limits _\Omega \mu }. \end{aligned}$$

### Proof

Using the uniform bound which is due to (B.8) in Remark B.3.

3.8 $$\begin{aligned} |\lambda ^\epsilon | + \int _\Omega |\nabla {\bar{u}}^\epsilon |^2\,dx \le \Vert K\Vert _\infty + \frac{m_0 \sup \limits _\Omega \mu }{\inf \limits _\Omega \mu }. \end{aligned}$$

Since $u^\epsilon$ is normalized by $\int _\Omega u^\epsilon \,dx = 0$, then

3.9 $$\begin{aligned} \sup _{{\varepsilon }>0}\Vert u^{\varepsilon }\Vert _{H^1({\Omega })}<\infty .\end{aligned}$$

Second, multiplying the first equation of (2.11) by $m^{\varepsilon }$, integrating by parts and using the second equation of (2.11), we obtain

$$\begin{aligned} \int _\Omega (m^{\varepsilon })^2=\lambda ^{\varepsilon }+\int _\Omega K m^{\varepsilon }-\frac{1}{2}\int _\Omega |{\nabla }_x u^{\varepsilon }|^2m_{\varepsilon }\le \Vert K\Vert _{L^\infty }+\lambda ^{\varepsilon }. \end{aligned}$$

Combining with (3.8), we deduce (3.7). $\square$

Thanks to (3.7), we may pass to a subsequence, and there exists $(\bar{\lambda }, {\bar{u}}, {\bar{m}}) \in \mathbb {R} \times H^1(\Omega )\times L^2(\Omega )$ such that

3.10 $$\begin{aligned} \lambda ^\epsilon \rightarrow \bar{\lambda }, \qquad u^\epsilon \overset{H^1}{\rightharpoonup }\ {\bar{u}},\quad \text { and }\quad m^\epsilon \overset{L^2}{\rightharpoonup }\ {\bar{m}}. \end{aligned}$$

### Proof of Theorem 3.4

Let $(\bar{\lambda }, {\bar{u}}, {\bar{m}}) \in \mathbb {R} \times H^1(\Omega )\times L^2(\Omega )$ be a subsequential limit as given by (3.10). Define the function

$$\begin{aligned} G_{\varepsilon }:C^2({\Omega })\times \mathcal P({\Omega })\ni (\phi ,m)\mapsto \int _{\Omega }(-{\varepsilon }\mu \Delta \phi +\frac{1}{2}|{\nabla }\phi |^2+(K-m^{\varepsilon }))dm, \end{aligned}$$

where in the case of $m \in L^1$, we follow the convention that $dm = m(x)dx$. First of all, by integrating the first equation of (2.11), we have

3.11 $$\begin{aligned} G_{\varepsilon }(u^{\varepsilon },m)=-\lambda ^{\varepsilon }=G_{\varepsilon }(u^{\varepsilon },m^{\varepsilon }) \quad \text { for all }m\in \mathcal P({\Omega })\cap C^2({\Omega }). \end{aligned}$$

Second, for any $\phi \in C^2({\Omega })$, define $z:=\phi -u^{\varepsilon }$. Then

$$\begin{aligned} G_{\varepsilon }(\phi ,m^{\varepsilon })-G_{\varepsilon }(u^{\varepsilon },m^{\varepsilon })&=\int _{\Omega }(-{\varepsilon }\mu \Delta z+\langle {\nabla }z,{\nabla }u^{\varepsilon }\rangle )dm^{\varepsilon }+\frac{1}{2}\int _{\Omega }|{\nabla }z|^2dm^{\varepsilon }\\&=\frac{1}{2}\int _{\Omega }|{\nabla }z|^2dm^{\varepsilon }\ge 0, \end{aligned}$$

where the second equality follows from multiplying the second equation of (2.11) by *z* and integrating by parts. We thus deduce that, for all $(\phi ,m)\in C^2({\Omega })\times \mathcal P({\Omega })$,

3.12 $$\begin{aligned} G_{\varepsilon }(u^{\varepsilon },m)\le G_{\varepsilon }(u^{\varepsilon },m^{\varepsilon })\le G_{\varepsilon }(\phi ,m^{\varepsilon }). \end{aligned}$$

Using

$$\begin{aligned} \int _{\Omega }(\bar{m})^2\le \underset{{\varepsilon }\rightarrow 0}{\lim \inf }\int _{\Omega }(m^{\varepsilon })^2, \end{aligned}$$

which is a consequence of Fatou’s lemma, we deduce that

3.13 $$\begin{aligned} \lim _{{\varepsilon }\rightarrow 0} G_{\varepsilon }(\phi ,m^{\varepsilon })\le \int _{\Omega }(\frac{1}{2}|{\nabla }\phi |^2+(K-\bar{m}))\bar{m} \quad \text { for all }\phi \in C^2({\Omega }). \end{aligned}$$

Furthermore, if $m\in \mathcal P({\Omega })\cap C^2({\Omega })$ is such that $\nu \cdot \nabla (\mu m) = 0$ on $\partial \Omega$,

3.14 $$\begin{aligned} \lim _{{\varepsilon }\rightarrow 0}G_{\varepsilon }(u^{\varepsilon },m)\ge \lim _{{\varepsilon }\rightarrow 0}\left( \int _{\Omega }(K-m^\epsilon )dm -\int _{\Omega }u^{\varepsilon }({\varepsilon }\Delta (\mu m))\right) =\int _{\Omega }(K-\bar{m})m.\nonumber \\ \end{aligned}$$

Here, we used $\int |\nabla u^\epsilon |^2\,dm \ge 0$ and integrated by parts. Hence, by taking $\phi = 1$ and combining (3.12), (3.13) and (3.14), we deduce that for any $m\in \mathcal P({\Omega })\cap C^2({\Omega })$ such that $\nu \cdot \nabla (\mu m) = 0$ on $\partial \Omega$, there holds

$$\begin{aligned} \int _{\Omega }(K-\bar{m})m\le -\lambda _0\le \int _{\Omega }(K-\bar{m})\bar{m}. \end{aligned}$$

By approximation, we observe that the above inequality holds for all $m \in \mathcal {P}(\Omega )$. This shows that $\bar{m}$ satisfies the definition of an IFD. The rest follows from Lemma 3.2 and the constraint $\int m^\epsilon \,dx = \bar{m}_0$ in (2.11).

### Proof of Theorem 3.5(a)

In this section, we establish the uniform convergence of the ergodic measure $m^\epsilon$ to the IFD as $\epsilon \rightarrow 0$, in the case of domains with dimension $n\ge 2$ assuming the stronger condition that $\mu$ is constant. By scaling in *x*, we may assume without loss of generality that $\mu \equiv \frac{1}{2},$ and (2.11) becomes

3.15 $$\begin{aligned} {\left\{ \begin{array}{ll} \lambda ^\epsilon - \frac{\epsilon }{2}\Delta u^\epsilon + \frac{1}{2} |\nabla u^\epsilon |^2 + F(x, m^\epsilon ) =0& \text { for }x \in \Omega ,\\ -\frac{\epsilon }{2} \Delta m^\epsilon - \textrm{div}\left( m^\epsilon {\nabla u^\epsilon }\right) =0 & \text { for }x\in \Omega ,\\ \nu \cdot \nabla u^\epsilon = \nu \cdot \nabla m^\epsilon = 0 & \text { for }x\in \partial \Omega ,\\ \int _\Omega u^\epsilon \,dx = 0 \quad \text { and }\quad \int _\Omega m^\epsilon \,dx = \bar{m}_0.& \end{array}\right. } \end{aligned}$$

Observe that the Fokker-Planck equation implies that $m^\epsilon (x)$ can be expressed as a *Boltzmann distribution* with Hamiltonian $u^\epsilon (x)$ and that a *partition function* $\bar{C}$, i.e.

3.16 $$\begin{aligned} m^\epsilon (x) = \bar{C} {\exp \left( \frac{-2 u^\epsilon (x)}{\epsilon }\right) }, \end{aligned}$$

where the constant $\bar{C} = \bar{m}_0 \left[ \int _\Omega \exp \left( \frac{-2 u^\epsilon (x)}{\epsilon }\right) \,dx\right] ^{-1}$ is chosen to ensure $\int _\Omega m^\epsilon \,dx = \bar{m}_0$. This follows from the fact that both $\exp \left( \frac{-2{\bar{u}}(x)}{\epsilon }\right)$ and $m^\epsilon$ are positive eigenfunctions of the same linear elliptic operator (corresponding to the zero eigenvalue), and must be linearly dependent, thanks to the Krein-Rutman Theorem (Kreĭn and Rutman 1950).

We take $\phi _\epsilon (x) = e^{-{u^\epsilon }/\epsilon }$ and seek to solve the nonlinear eigenvalue problem

3.17 $$\begin{aligned} {\left\{ \begin{array}{ll} \epsilon ^2 \Delta \phi _\epsilon + 2\left( F\left( x, \phi ^2_\epsilon \right) + \bar{\lambda }\right) \phi _\epsilon = 0 & \text { in }\Omega ,\\ \int _\Omega \phi ^2_\epsilon \,dx = \bar{m}_0 \quad \text { and }\quad n \cdot \nabla \phi _\epsilon & \text { on }\partial \Omega . \end{array}\right. } \end{aligned}$$

To solve (3.17), we consider, for each $\epsilon >0$ and $\Lambda \in \mathbb {R}$, the following semilinear equation

3.18 $$\begin{aligned} {\left\{ \begin{array}{ll} {\epsilon ^2} \Delta w + 2\left( F(x,w^2) + \Lambda \right) w = 0 & \text { in }\Omega ,\\ n \cdot \nabla w =0 & \text { on }\partial \Omega . \end{array}\right. } \end{aligned}$$

#### Proposition 3.7

For each $\epsilon >0$ and $\Lambda >\underline{\Lambda }_\epsilon$, (3.18) has a unique positive solution $w_{\epsilon ,\Lambda }$, where $\underline{\Lambda }_\epsilon$ is the principal eigenvalue of

3.19 $$\begin{aligned} {\left\{ \begin{array}{ll} \epsilon ^2 \Delta \varphi + 2\left( F(x,0)+ \underline{\Lambda }\right) \varphi = 0 & \text { in }\Omega ,\\ n \cdot \nabla \varphi =0 & \text { on }\partial \Omega . \end{array}\right. } \end{aligned}$$

Furthermore,

- $$w_{\epsilon ,\Lambda }(x) < w_{\epsilon ,\Lambda '}(x)$$ in $$\bar{\Omega }$$ if $$\Lambda < \Lambda '.$$
- $$w_{\epsilon ,\Lambda } \searrow 0$$ as $$\Lambda \searrow \underline{\Lambda }_\epsilon $$.
- There exists $\overline{\Lambda }>0$ independent of $\epsilon$ such that $\int _\Omega |w_{\epsilon ,\Lambda }|^2 \,dx > m_0$ for any $\epsilon >0$ and $\Lambda \in [\overline{\Lambda },\infty )$.

#### Proof

Fix an arbitrary $\epsilon >0$. The existence of $w_{\epsilon ,\Lambda }$ for $\Lambda > \underline{\Lambda }_\epsilon$ is classical (Cantrell and Cosner 2003; Lam and Lou 2022). For (a), observe that for $\Lambda '> \Lambda$, $w_{\epsilon , \Lambda '}$ is a strict supersolution of (3.18), so it follows by comparison (Lam and Lou 2022, Corollary 5.1.9) that $w_{\epsilon ,\Lambda } < w_{\epsilon ,\Lambda '}$ in $\bar{\Omega }$. This proves (a).

Thanks to (a), the family $\{w_{\epsilon ,\lambda }\}_{ \Lambda \in (\underline{\Lambda }_\epsilon , \underline{\Lambda }_\epsilon +1]}$ is bounded in $L^\infty (\Omega )$. It follows by elliptic $L^p$ estimates that this family is bounded in $W^{2,p}(\Omega )$ for any $p >1$. By passing to a subsequence, we may assume that $w_{\epsilon ,\Lambda } \rightarrow {\bar{w}}$ as $\Lambda \searrow \underline{\Lambda }_\epsilon$ weakly in $W^{2,p}(\Omega )$. Moreover, the limit ${\bar{w}}$ satisfies

3.20 $$\begin{aligned} \epsilon ^2 \Delta {\bar{w}} + 2(F(x,0) + \underline{\Lambda }_\epsilon ){\bar{w}} = 2(F(x,0) - F(x,{\bar{w}}^2))\ge 0 \quad \text { in }\Omega \end{aligned}$$

and the Neumann boundary condition on $\partial \Omega$.

Next, multiplying both sides of (3.20) by principal eigenfunction $\varphi _\epsilon >0$ of (3.19) and integrating by parts, we deduce that

$$\begin{aligned} 0 = \int _\Omega \varphi _\epsilon (F(x,0) - F(x,{\bar{w}}^2)) \,dx \ge 0. \end{aligned}$$

Since $s\mapsto F(x,0)$ is strictly decreasing, it follows that $\bar{w} \equiv 0$. This proves (b).

For (c), choose $\overline{\Lambda }= \sup _\Omega \left( - F(x, |\bar{m}_0|^2/|\Omega |)\right) +1$. Then one obtains that for each $\Lambda \in [\overline{\Lambda },\infty )$ $\underline{w} = |\Omega |^{-1/2}\bar{m}_0 >0$ is a strict subsolution of (3.18). It follows from the comparison principle ( Lam and Lou (2022), Corollary 5.1.9) that

$$\begin{aligned} w_{\epsilon ,\Lambda } \ge |\Omega |^{-1/2}m_0 \quad \text { in }~\Omega ~\text { for }~ \Lambda \in [\overline{\Lambda }, \infty ). \end{aligned}$$

This proves (c). $$\square $$

#### Proposition 3.8

For each $\epsilon >0$, the nonlinear eigenvalue problem (3.17) has a unique solution $(\lambda _\epsilon ,\phi _\epsilon )$.

#### Proof

Let $\epsilon >0$ be fixed. By Lemma 3.7, there exists a unique $\Lambda$ such that $\int _\Omega w_{\epsilon , \Lambda }^2 \,dx = 1$. It follows that $(\lambda _\epsilon ,\phi _\epsilon )=(\Lambda ,w_{\epsilon ,\Lambda })$ exists, and is uniquely determined. $\square$

#### Proof of Theorem 3.5(a)

For $j \in \mathbb {N}$, let $(\lambda _j, {u}_j, {m}_j)$ be the solution of (3.15) with $\epsilon = \epsilon _j \rightarrow 0$. By Lemma 3.7, $\lambda _j$ is the unique number in $(\underline{\Lambda }_\epsilon , \overline{\Lambda })$ such that

$$\begin{aligned} \int _\Omega |w_j|^2\,dx = \bar{m}_0 \quad \text { where } w_j = w_{\epsilon _j, \lambda _j}, \end{aligned}$$

and that

$$\begin{aligned}({u}_j, {m}_j) = (-\epsilon \log w_j , w_j^2).\end{aligned}$$

Next, we claim that

$$\begin{aligned} |\lambda _j| + \sup _\Omega |w_j| \le C \quad \text { for some }C \text { independent of }j. \end{aligned}$$

Indeed, $\lambda _j\in (\underline{\Lambda }_\epsilon , \overline{\Lambda })$ is uniformly bounded from above by Proposition 3.7(c). Also, since the lower bound satisfies $\underline{\Lambda }_\epsilon \rightarrow -\sup _\Omega F(\cdot ,0)$ as $\epsilon \rightarrow 0$ (see, e.g. Lam and Lou 2022, Proposition 1.3.16), it follows that $\{\lambda _j\}$ is uniformly bounded.

Since $\sup _\Omega F(\cdot ,M_0) \rightarrow -\infty$ as $M_0 \rightarrow +\infty$, we can obtain that $w = w_{\epsilon _j, \lambda _j}$ is also bounded from above. Indeed, let $\overline{\Lambda }_2$ be an upper bound of $\lambda _j$, and choose $M_2\ge 1$ such that $F(x,M_2) + \overline{\Lambda }_2 \le 0$, then one can argue similarly as Lemma 3.7(c) that $w_{\epsilon _j, \lambda _j} \le M_2$. This means ${m}_j = w_{\epsilon _j, \lambda _j} ^2$ is bounded from above uniformly in *j*.

Since $\{\lambda _j\}$ is a bounded sequence, we may pass to a further subsequence and assume that $\lambda _j \rightarrow \bar{\lambda }$. It follows that, for each $\delta >0$,

3.21 $$\begin{aligned} \lim _{\epsilon _j \rightarrow 0} w_{\epsilon _j, \bar{\lambda }- \delta } \le \liminf _j w_j \le \limsup _j w_j \le \lim _{\epsilon _j \rightarrow 0} w_{\epsilon _j, \bar{\lambda }+ \delta }. \end{aligned}$$

By ( Lam and Lou (2022), Theorem 5.2.5), it holds that

$$\begin{aligned} \lim _{\epsilon _j \rightarrow 0}w_{\epsilon _j, \bar{\lambda }+ \delta } = \max \{0, K(x) + \bar{\lambda }\pm \delta \} \quad \text { uniformly in }\bar{\Omega }. \end{aligned}$$

Hence, (3.21) becomes

$$\begin{aligned} \max \{0, K(x) +\bar{\lambda }- \delta \} \le \liminf _j w_j \le \limsup _j w_j \le \max \{0, K(x) + \bar{\lambda }+ \delta \} \end{aligned}$$

uniformly in $\bar{\Omega }$. Since $\delta >0$ is arbitrary, we may let $\delta \searrow 0$. This proves the convergence of $m_j \rightarrow \max \{K(x) - \bar{\lambda },0\}$. Using the constraint $\int _\Omega m_j\,dx = {\bar{m}}_0$, we deduce that the limit value $\bar{\lambda }$ is independent of subsequence, and so the convergence holds for the full limit as $\epsilon \rightarrow 0$. This concludes the proof. $\square$

### Proof of Theorem 3.5(b)

By the bounds in Remark B.3, we can pass to a subsequence and suppose

3.22 $$\begin{aligned} \lambda ^\epsilon \rightarrow \bar{\lambda }\quad \text { and }\quad u^\epsilon \rightarrow {\bar{u}} \quad \text { weakly in }H^1. \end{aligned}$$

Since $\mu (x)$ is nonconstant, $m^\epsilon$ can no longer be solved explicitly in terms of $u^\epsilon$ as in (3.16). We need the following lemma instead.

#### Lemma 3.9

Fix $$\epsilon >0$$.

- Suppose $m^\epsilon > {\bar{K}} + \lambda ^\epsilon$ in (*a*, *b*), then $\mu m^\epsilon$ cannot have a local maximum in (*a*, *b*).
- Suppose $m^\epsilon < {\bar{K}} + \lambda ^\epsilon$ in (*a*, *b*), then $\mu m^\epsilon$ cannot have a local minimum in (*a*, *b*).

#### Proof

We prove (a), and omit the proof of (b) as can be proved in a similar manner.

First, by the no-flux boundary condition, we may integrate the second equation of the ergodic problem (2.11) (under the assumption that $\Omega =(0,1)$) from 0 to *x* to obtain

3.23 $$\begin{aligned} \epsilon (\mu m^\epsilon )_x = - m^\epsilon u_x \end{aligned}$$

Next, observe from the first equation of (2.11) that $u^\epsilon$ cannot have a local minimum in (*a*, *b*). Therefore, either $u^\epsilon$ is strictly monotone in (*a*, *b*), or there exists $c \in (a,b)$ such that $u^\epsilon$ is strictly increasing in (*a*, *c*) and strictly decreasing in (*c*, *b*). The conclusion follows from (3.23). $\square$

#### Lemma 3.10

(Uniform $L^\infty$ upper bound of *m*)

3.24 $$\begin{aligned} 0\le \mu m^\epsilon \le \Vert \mu ( K + \lambda ^\epsilon )\Vert _{L^\infty (\Omega )} + \frac{\bar{m}_0}{|\Omega |} \qquad \text { for all }x\in \Omega .\end{aligned}$$

#### Proof

Since $|m^\epsilon |_{L^1}\le C$, Chebyshev’s inequality says that

$$\begin{aligned} \inf _{I} m^\epsilon \le \frac{1}{|I|}|m^\epsilon |_{L^1} \quad \text { for any interval }I. \end{aligned}$$

In particular $\inf \limits _\Omega m^\epsilon < \bar{m}_0 / |\Omega |$ and the inequality (3.24) holds for some $x_0 \in \Omega$.

Suppose the conclusion is false, and we choose a maximal interval $I_\epsilon = (a_\epsilon , b_\epsilon )$ in which $\mu (x) m^\epsilon (x) > \Vert \mu (K + \lambda ^\epsilon )\Vert _\infty + \frac{{\bar{m}}_0}{|\Omega |}$. We divide into two cases:

$$\begin{aligned} \mathrm{(i)}\quad \{a_\epsilon , b_\epsilon \} \cap \partial \Omega = \emptyset , \quad \text { and } \quad \mathrm{(ii)} \quad \{a_\epsilon , b_\epsilon \} \cap \partial \Omega \ne \emptyset . \end{aligned}$$

In the former case, we may choose a point $y_\epsilon$ and an open interval $I_\epsilon \ni y_\epsilon$ such that (i) $\mu (x)m^\epsilon (x) > \Vert \mu (\bar{K} + \lambda ^\epsilon )\Vert _\infty$ in ${\bar{I}}_\epsilon$ and (ii) $\mu (x)m^\epsilon (x)$ attains local maximum at a point $y_\epsilon \in \textrm{Int}\,I_\epsilon$. However, $u^\epsilon$ does not have a local minimum point in $I_\epsilon$ by the maximum principle. It follows from (3.23) that $\mu m^\epsilon$ does not attain local max in $I_\epsilon$. This is a contradiction.

In the latter case, then exactly one of the boundary point (say, $a_\epsilon$) of $I_\epsilon$ belongs to the boundary of $\Omega$, since $I_\epsilon \ne \Omega$. One can extend the problem by reflecting at the boundary point $a_\epsilon \in \partial \Omega$ to obtain an interior local maximum point $y_\epsilon$, and then argue as in case (i) to derive a contradiction. $\square$

#### Lemma 3.11

Suppose that there is $\delta >0$ and sequences $\epsilon = \epsilon _k \rightarrow 0$ and $I=(a,b)$ such that

$$\begin{aligned} m^{\epsilon _k} - K -\lambda ^{\epsilon _k} \ge \delta ^2 \quad \text { in }I, \end{aligned}$$

then

$$\begin{aligned} m^\epsilon \rightarrow 0 \quad \text { in }C_{loc}(I).\end{aligned}$$

#### Proof

Fix a small $\eta >0$, we need to show that

3.25 $$\begin{aligned} m^\epsilon \rightarrow 0 \quad \text { in }[a+\eta ,b-\eta ]. \end{aligned}$$

The function $u^\epsilon$, with $\epsilon = \epsilon _k$, satisfies

3.26 $$\begin{aligned} -\epsilon \mu u^\epsilon _{xx} + |u^\epsilon _x|^2 \ge \delta ^2 \quad \text { in }I. \end{aligned}$$

Thanks to (3.22), and that $H^1 \subset C^{1/2}$, $u^\epsilon \rightarrow {\bar{u}}$ uniformly. It is standard to see that the uniform limit ${\bar{u}}$ is a viscosity supersolution (Barles 2013) of

3.27 $$\begin{aligned} {\left\{ \begin{array}{ll} |w_x|^2 = \delta ^2 \quad \text { in }(a,b),\\ w(a) = {\bar{u}}(a),\quad w(b) = {\bar{u}}(b). \end{array}\right. } \end{aligned}$$

By the maximum principle, $u^\epsilon$ and its limit ${\bar{u}}$ cannot attain a local minimum in (*a*, *b*), so there exists ${\bar{x}} \in [a,b]$ such that

3.28 $$\begin{aligned} \bar{u}_x \ge 0 \quad \text {a.e. in }(a,{\bar{x}}), \quad \text { and }\quad {\bar{u}}_x \le 0 \quad \text { a.e. in } ({\bar{x}},b). \end{aligned}$$

(We regard $(a,{\bar{x}})$ as empty when ${\bar{x}} = a$ and a similar convention holds for $({\bar{x}}, b)$.) Note that $\bar{u}_x \in L^2$ is defined almost everywhere.

Next, note that (3.27) has a unique viscosity solution

$$\begin{aligned} w(x) = \min \{{\bar{u}}(a) + \delta x, {\bar{u}}(b) + \delta (b-x)\} \quad \text { for }x \in (a,b). \end{aligned}$$

It follows by comparison that

$$\begin{aligned} \bar{u}(x) \ge \min \{{\bar{u}}(a) + \delta (x-a), {\bar{u}}(b) + \delta (b-x)\}\quad \text { for }x \in (a,b). \end{aligned}$$

Rearranging (3.23), we have

3.29 $$\begin{aligned} [\log (\mu m^\epsilon )]_x = - \frac{ (u^\epsilon )_x}{\epsilon \mu }. \end{aligned}$$

Integrating again, we have

3.30 $$\begin{aligned} \log \frac{\mu (x)m^\epsilon (x)}{\mu (y)m^\epsilon (y)} = -\frac{1}{\epsilon } \int _y^x \frac{(u^\epsilon )_x(z)}{\mu (z)}\,dz = -\frac{1}{\epsilon } \left[ \int _y^x \frac{{\bar{u}}_x(z)}{\mu (z)}\,dz + o(1)\right] . \end{aligned}$$

where we used $u^\epsilon \rightarrow {\bar{u}}$ weakly in $H^1$.

By (3.28), ${\bar{u}}_x$ does not change sign in $(a,{\bar{x}})$ (resp. $({\bar{x}},b)$), and it follows that

3.31 $$\begin{aligned} - \log \frac{\mu (x)m^\epsilon (x)}{\mu (y)m^\epsilon (y)}\ge &  \frac{1}{\epsilon \max \mu } \left( \int _y^x{\bar{u}}_x(z)\,dz + o(1)\right) \nonumber \\= &  \frac{1}{\epsilon \max \mu } ({\bar{u}}(x) - {\bar{u}}(y) + o(1)) \end{aligned}$$

for $a \le y < x \le {\bar{x}}$. Setting $y=a$, we obtain

$$\begin{aligned} {\mu (x) m^\epsilon (x)}&\le C|m^\epsilon |_{L^\infty } \exp \left( -\frac{{\bar{u}}(x) - {\bar{u}}(a)+ o(1)}{\epsilon \max \mu } \right) \\&\le C|m^\epsilon |_{L^\infty } \exp \left( -\frac{\delta (x-a)+ o(1)}{\epsilon \max \mu } \right) \quad \text { for } x \in [a + \eta ,{\bar{x}}]. \end{aligned}$$

If ${\bar{x}} = b$, then we are done, if not, we argue similarly in the interval $({\bar{x}},b-\eta )$ to obtain

$$\begin{aligned} {\mu (x) m^\epsilon (x)} \le C|m^\epsilon |_{L^\infty } \exp \left( -\frac{\delta (b-x) + o(1)}{\epsilon \max \mu } \right) \quad \text { for }x \in [{\bar{x}},b - \eta ]. \end{aligned}$$

Since $\inf \mu >0$ and $m^\epsilon$ is bounded in $L^\infty$ (thanks to Lemma 3.10), we proved that $m^\epsilon \rightarrow 0$ uniformly in each compact subset of (*a*, *b*). $\square$

We record the following observation from the proof of Lemma 3.11.

#### Corollary 3.12

Suppose $|{\bar{u}}_x|^2 \ge \delta ^2$ in (*a*, *b*) in viscosity sense, then

$$\begin{aligned}m^\epsilon \rightarrow 0 \quad \text { in }C_{loc}((a,b)).\end{aligned}$$

#### Proof of Theorem 3.5(b)

Passing to a sequence, we may assume that (3.22) holds for some $\bar{\lambda }\in \mathbb {R}$ and ${\bar{u}} \in H^1$. It remains to prove that $\bar{\lambda }$ is uniquely determined by $\int \max \{{\bar{K}} + \bar{\lambda },0\}\,dx = m_0$, and that

3.32 $$\begin{aligned} m^\epsilon (x) \rightarrow \max \{ K(x) +\bar{\lambda },0\} \quad \text { uniformly as }\epsilon \rightarrow 0. \end{aligned}$$

**Step** **#1**. $m^\epsilon \rightarrow 0$ in $C_{loc}(I_-)$, where $I_-=\{x:~ K(x) + \bar{\lambda }<0\}$.

For each closed interval $[a,b] \subset I_-$, choose $[a', b']$ such that

$$\begin{aligned} [a,b] \subset (a',b') \quad \text { and }\quad [a',b'] \subseteq I_-. \end{aligned}$$

It follows by definition of $I_-$ that there exists $\underline{\delta }^2 >0$ such that for any $0<\epsilon \ll 1$,

$$\begin{aligned} m^\epsilon - K - \lambda ^\epsilon \ge \underline{\delta }^2 \quad \text { in }(a',b'). \end{aligned}$$

It follows from Lemma 3.11 that $m^\epsilon \rightarrow 0$ uniformly in compact subsets of $(a',b')$, i.e. $m^\epsilon \rightarrow 0$ uniformly in [*a*, *b*]. This proves $m^\epsilon \rightarrow 0$ in $C_{loc}(I_-)$.

**Step #2.** For each $\delta >0$ and $\eta >0$, there exists $\epsilon _0= \epsilon _0(\delta ,\eta )>0$ such that for any $\epsilon \in (0,\epsilon _0]$, the inequality

3.33 $$\begin{aligned} \inf _I (m^\epsilon - K - \lambda ^\epsilon ) <\delta \end{aligned}$$

holds uniformly for all interval $I= (a,b) \subset \{x:~K + \bar{\lambda }\ge -\delta /2\}$ such that $|b-a| \ge 2\eta$.

Suppose not, then there exist $\eta ,\delta >0$ and $I_\epsilon =(a_\epsilon ,b_\epsilon )$ such that

3.34 $$\begin{aligned} I_\epsilon \subset \{x:~K + \bar{\lambda }\ge -\delta /2\}, \qquad b_\epsilon - a_\epsilon >2\eta ,\quad \text { and }\quad \inf _{(a_\epsilon ,b_\epsilon )} (m^\epsilon - K - \lambda ^\epsilon ) \ge \delta .\nonumber \\ \end{aligned}$$

Without loss, we may assume that $a_\epsilon \rightarrow a$ and $b_\epsilon \rightarrow b$ for some $a \ne b$, such that $a < b$. Then, $u^\epsilon (x) \rightarrow {\bar{u}}$ uniformly and ${\bar{u}}$ satisfies, in viscosity sense,

$$\begin{aligned} |{\bar{u}}_x|^2 \ge \delta \quad \text { in }(a,b). \end{aligned}$$

By Corollary 3.12, we deduce that, as $\epsilon \rightarrow 0$,

$$\begin{aligned} m^\epsilon (x) \rightarrow 0 \quad \text { in }C_{loc}((a,b)). \end{aligned}$$

However, this contradicts with the fact that

$$\begin{aligned} \frac{\delta }{2} \le \delta + K + \lambda ^\epsilon \le m^\epsilon \quad \text { at }x = \frac{a+b}{2} \in (a_\epsilon ,b_\epsilon ) \cap (a,b). \end{aligned}$$

This completes Step #2.

**Step #3.** For each $\delta >0$, there exists $\epsilon _1= \epsilon _1(\delta )>0$ such that for any $\epsilon \in (0,\epsilon _1]$, we have

3.35 $$\begin{aligned} \sup _{\{K(x) + \bar{\lambda }\ge -\delta /4\}} (m^\epsilon - K - \lambda ^\epsilon ) \le 2\delta . \end{aligned}$$

Suppose not, then there exists a sequence $\epsilon =\epsilon _j \rightarrow 0$ and $c_\epsilon \in \{K(x) + \bar{\lambda }\ge -\delta /4\}$ such that $(m^\epsilon - K - \lambda ^\epsilon )(c_\epsilon ) > \delta$. Since $c_\epsilon$ is uniformly bounded away from $\{K(x) + \bar{\lambda }< -\delta /2\}$, we can use the previous step to deduce that there exist $\tilde{\delta }$, $a_\epsilon< c_\epsilon < b_\epsilon$ such that

$$\begin{aligned} (a_\epsilon , b_\epsilon ) \subset \{K(x) + \bar{\lambda }\ge -\delta /2\}, \qquad b_\epsilon -a_\epsilon \rightarrow 0 \end{aligned}$$

and

$$\begin{aligned} {\left\{ \begin{array}{ll} m^\epsilon - K - \lambda ^\epsilon = \delta & \text { at }x \in \{a_\epsilon ,b_\epsilon \},\\ m^\epsilon - K - \lambda ^\epsilon>\delta & \text { in }(a_\epsilon ,b_\epsilon ),\\ m^\epsilon - K - \lambda ^\epsilon >2\delta & \text { at }x=c_\epsilon . \end{array}\right. } \end{aligned}$$

Without loss of generality, we may assume that $0<b_\epsilon -a_\epsilon <\eta$, where $\eta >0$ is small enough such that

3.36 $$\begin{aligned} &  |\mu (x)K(x)- \mu (y) K(y)|< \frac{\delta \inf _\Omega \mu }{2},\quad \nonumber \\ &  |\mu (x)- \mu (y) |< \frac{\delta \inf _\Omega \mu }{2(|\bar{\lambda }| + \delta )} \quad \text { whenever }|x-y| < \eta . \end{aligned}$$

This choice yields

3.37 $$\begin{aligned} \inf _I \left[ \mu (K + \bar{\lambda }+ 2\delta )\right] > \sup _{I} \left[ \mu (K + \bar{\lambda }+ \delta )\right] \end{aligned}$$

for any interval *I* containing *x* of length smaller than $\eta$. Hence,

$$\begin{aligned} \mu (c_\epsilon )m^\epsilon (c_\epsilon )> &  \mu (c_\epsilon )(K(c_\epsilon ) + \lambda ^\epsilon + 2\delta ) > \sup _{(a_\epsilon ,b_\epsilon )}\mu (K + \lambda ^\epsilon + \delta )\\ \ge &  \max \{\mu (a_\epsilon )m(a_\epsilon ),\mu (b_\epsilon )m(b_\epsilon )\}. \end{aligned}$$

This means that $\mu m$ has an interior local maximum at some $c'_\epsilon \in (a_\epsilon ,b_\epsilon )$ such that $m^\epsilon - K - \lambda ^\epsilon >0$ at $x=c'_\epsilon$. This is a contradiction with Lemma 3.9. This completes Step #3. Combining Steps #1 and #3, we deduce that

3.38 $$\begin{aligned} \limsup \limits _{\epsilon \rightarrow 0} m^\epsilon (x) \le \max \{0, K(x) + \bar{\lambda }\} \quad \text { uniformly in }\Omega . \end{aligned}$$

**Step #4.** We claim that for each $\delta >0$,

$$\begin{aligned} \sup _I (m^\epsilon - K - \lambda ^\epsilon ) \ge - \frac{4 (1 + \Vert {\bar{u}}\Vert _\infty ) \epsilon }{\delta ^2} \end{aligned}$$

for any interval $I=(x_0 - \delta ,x_0 + \delta )$ in $\Omega$.

Indeed, by the uniform convergence $u^\epsilon \rightarrow {\bar{u}}$, we can set $C_2 = 1 + \Vert {\bar{u}}\Vert _\infty$ to ensure that

$$\begin{aligned} \Vert u^\epsilon \Vert _\infty< C_2 \quad \text { for all } 0 < \epsilon \ll 1. \end{aligned}$$

Fix an arbitrary $x_0$ and let $\phi = 2C_2\left( \frac{x-x_0}{\delta }\right) ^2$. Then $\phi (x_0 \pm \delta ) = 2C_2$ implies that

$$\begin{aligned} \sup _{I\cap \Omega } (u^\epsilon - \phi ) \ge (u^\epsilon - \phi )(x_0) > -C_2. \end{aligned}$$

Note that for $y \in \partial (I\cap \Omega )$, we either have (i) $y=x_0 \pm \delta$ or (ii) $y \in \partial \Omega$.

In case (i), $(u^\epsilon - \phi ) (y) < -C_2$. In case (ii), the outer normal derivative of $u^\epsilon - \phi$ is strictly negative since $(u^\epsilon )_x (y) =0$. In both cases, we conclude that $u^\epsilon - \phi$ has a local maximum at some interior point $y_\epsilon \in I \cap \Omega$, where it holds that $(u^\epsilon )''(y_\epsilon ) \le \phi ''(y_\epsilon )$ and $(u^\epsilon )'(y_\epsilon ) = \phi '(y_\epsilon )$. Thus, by the first equation of (2.11),

$$\begin{aligned} - \frac{4C_2 \epsilon }{\delta ^2} + \frac{|4 C_2|^2}{\delta ^4}|y_\epsilon - x_0|^2 \le m(y_\epsilon ) - K(y_\epsilon ) - \lambda ^\epsilon . \end{aligned}$$

This implies $\sup _{(x_0-\delta ,x_0+\delta )} (m^\epsilon - K - \lambda ^\epsilon ) \ge - \frac{4 C_2\epsilon }{\delta ^2}$. This completes Step #4.

**Step #5.** For each $\tilde{\delta }>0$, there exists $\epsilon _1= \epsilon _1(\tilde{\delta })>0$ such that for any $\epsilon \in (0,\epsilon _1]$, we have

3.39 $$\begin{aligned} \inf _\Omega (m^\epsilon - K - \lambda ^\epsilon ) \ge -2\tilde{\delta }. \end{aligned}$$

Suppose the claim does not hold, then there exists $\tilde{\delta }>0$ and $c_\epsilon \rightarrow c_0$ such that $(m^\epsilon - K - \lambda ^\epsilon )(c_\epsilon ) < -2\tilde{\delta }$. We may assume without loss that $c_0 \in \textrm{Int}\,\Omega$ (otherwise $c_0 \in \partial \Omega$ and we may extend the problem by reflection). Hence, by Step #4, there exists $a_\epsilon< c_\epsilon < b_\epsilon$ such that

$$\begin{aligned} b_\epsilon -a_\epsilon \rightarrow 0 \quad \text { and }\quad {\left\{ \begin{array}{ll} m^\epsilon - K - \lambda ^\epsilon =- \tilde{\delta }& \text { at }x \in \{a_\epsilon ,b_\epsilon \},\\ m^\epsilon - K - \lambda ^\epsilon<-\tilde{\delta }& \text { in }(a_\epsilon ,b_\epsilon ),\\ m^\epsilon - K - \lambda ^\epsilon <-2\tilde{\delta }& \text { at }x=c_\epsilon . \end{array}\right. } \end{aligned}$$

Again, we can assume $b_\epsilon -a_\epsilon < \eta$ for some $\eta >0$ small enough so that

$$\begin{aligned} \sup _I \left[ \mu (K + \bar{\lambda }+ 2\tilde{\delta })\right] < \inf _I \left[ \mu (K + \bar{\lambda }+ \tilde{\delta })\right] \end{aligned}$$

holds for every interval with length smaller than $\eta$. Hence, we obtain again

$$\begin{aligned} \mu (c_\epsilon )m^\epsilon (c_\epsilon )< &  \mu (c_\epsilon )(K(c_\epsilon ) + \lambda ^\epsilon + 2\tilde{\delta }) < \inf _{(a_\epsilon ,b_\epsilon )}\mu (K + \lambda ^\epsilon + \tilde{\delta })\\\le &  \min \{\mu (a_\epsilon )m(a_\epsilon ),\mu (b_\epsilon )m(b_\epsilon )\}. \end{aligned}$$

This means that $\mu m$ has an interior local minimum at some $c'_\epsilon \in (a_\epsilon ,b_\epsilon )$ such that $m^\epsilon - K - \lambda ^\epsilon <0$ at $x=c'_\epsilon$. This is a contradiction with Lemma 3.9. In particular, we establish in this step that

3.40 $$\begin{aligned} \liminf _{\epsilon \rightarrow 0} m^\epsilon (x) \ge K(x) + \bar{\lambda }\quad \text { uniformly in }\Omega . \end{aligned}$$

Combining (3.38) and (3.40), and the nonnegativity of $m^\epsilon$, we prove that $m^\epsilon \rightarrow \max \{K(x) + \bar{\lambda },0\}$ uniformly in $\Omega$. By the integral constraint of $m^\epsilon$, it follows that $\bar{\lambda }$ is uniquely determined and we may conclude the proof of Theorem 3.5 as before. $\square$

## Discussion

By now, the ideal free distribution (IFD) (Fretwell and Lucas 1969) is a well established concept in ecological theory, and it has many ramifications in the evolution of dispersal (Cosner 2014). At the basic level, the IFD is derived in a static setting as the Nash equilibrium of the habitat selection game, and it has been demonstrated that it is an evolutionarily stable strategy in the adaptive dynamics framework (Averill et al. 2012; Cantrell et al. 2010). In this paper, we leverage the framework of mean field games (MFG), introduced by Lasry and Lions recently, to give an alternative derivation of the IFD in a dynamic setting. Mean field game (MFG) models, proposed by Lasry and Lions (2007) and Huang et al. (2006) independently, are a set of PDEs used to approximate an infinite number of players behaving as a Nash equilibrium with respect to the differential game. In contrast to existing models where (usually two) populations with prescribed dispersal strategies are allowed to compete (Hastings 1983; Dockery et al. 1998; Cantrell et al. 2010), MFG grants the individual the ability to optimize their performance as measured by a suitable payoff functional which is perturbed by the mean field term representing the average behavior of the infinite number of agents.

### Model assumptions and generalizations

An important feature in the MFG setting of this paper is that there is no birth or death in the model, so that having zero diffusion does not mean that the population can achieve IFD, and is therefore different from the setting in Cantrell et al. (2007). When the parameter $\epsilon >0$ (which appears originally in the cost functional $\mathcal {J}$) is small, then the cost of control becomes small and the drift due to control dominates over the standard noise due to diffusion in the Fokker-Planck equation governing the population density $m^T(t,x)$. It is this combination of large and optimal drift and a bounded diffusive movement that together enables the ideal free distribution.

In our model, the cost of motion is taken to be quadratic in the velocity for simplicity and for consistency with kinetic energy. If this assumption is relaxed to a more general form of convex function $L(v)\ne \frac{1}{2}v^2$, then it is no longer natural to work with $H^1$ estimates of the value function *u*. Nonetheless, we conjecture that an analogous argument holds.

The choice of fitness function $F(x,s) = K(x) - s$ can be significantly relaxed. In general, the same conclusions hold for any fitness function *F* satisfying (F2) in Section 2.1.

Regarding the fact that Theorem 3.5 requires $\Omega$ to be one-dimensional in case $\mu$ is nonconstant and hence is more restrictive than Theorem 3.4, it is of course natural to wonder whether some stronger (typically uniform) convergence might hold in higher dimensions. At the moment, we do not know whether it is possible to strengthen the result, as this hinges on quite involved technical aspects of ergodic mean field games. First of all, observe that Theorem 3.4 does not give any information regarding the convergence of the value function $u^\epsilon$, as this would require a priori estimates that are out of reach in higher dimensions. Second, such stronger estimates would be necessary to obtain better convergence of $m^\epsilon$. The underlying reason has to do with the lack of regularisation effect of the local coupling term $F(m) = K - m$; indeed, the usual setting considered in ergodic MFG assumes that *F* takes values in a Hölder space (e.g., $F = F(\rho * m)$ where $\rho$ is a smoothing kernel), which allows one to obtain, for instance, uniform Lipschitz regularity of $u^\epsilon$. We refer, for instance, to Cardaliaguet (2013), and we leave this question as an interesting problem.

The access to and use of information by the individual is critical in achieving IFD. In this work, the individual moves according to the gradient of value function, which is a special form of usage of full space-time information of the environment and of the overall population dynamics. In general, this can be considered as a approximation when each individual retains information of past realized fitness. Such information can for example result from personal experience or can be communicated from conspecifics.

### Related work

In Bondesan et al. (2025) the evolution of size distribution in a prey-predator model was considered. These models incorporates birth and death dynamics, while most papers in mean field games ignore such effects, except some recent work on the mean field games of branching processes (Claisse et al. 2023).

In Cardaliaguet et al. (2015), the MFG with degenerate parabolic operators was considered. In their setting, a weak notion of solution is introduced for the first order MFG, and the existence, uniqueness and stability of such weak solutions is proved via the connection with two optimization problems. Their work naturally encompasses the vanishing viscosity limit in a rather weak and sophisticated setting. In particular, they demonstrated an exponential rate of convergence of the solutions to the time-dependent problem to those of the ergodic problem which is uniform away from initial and terminal time ( Cardaliaguet and Porretta (2020), Theorem 1.14). In contrast, our main focus is the vanishing viscosity limit for classical solutions of the ergodic problem itself $(\lambda ^\epsilon , u^\epsilon , m^\epsilon ) \rightarrow (\bar{\lambda },{\bar{u}}, {\bar{m}})$. We provide conditions for uniform convergence, and emphasize the connection with the game theoretical interpretation of the uniform limits $(\bar{\lambda },{\bar{u}}, {\bar{m}})$ which is its connection with the ideal free distribution of players.

In this work, we mainly considered the control of the drift of the diffusion process governing the movement of agents. We expect that the ideal free distribution can also arise from other modes of control (as the cost of control tends to zero), such as the control of diffusion rate ( Fleming and Soner (2006), Chapter IV), and the optimal switching between movement behaviors (Pham 2009, Chapter 5). It will also be interesting to consider the convergence to IFD when the payoff function is periodically varying in time (Cantrell and Cosner 2018; Cantrell et al. 2021); in such a case, we expect that the population of players will converge to the state where the fitness is equilibrated in space, but not necessarily in time.

## Data Availability

No new data were generated or analyzed in support of this research.

## Proof of Proposition 2.1

In this section, we consider the convergence of classical solutions $(u^T,m^T): [0,T]\times \Omega \rightarrow \mathbb {R}^2$ to the finite horizon MFG (2.10) to the solutions $(\lambda ,{\bar{u}}, \bar{m})): \Omega \rightarrow \mathbb {R}^2$ to the ergodic MFG (2.11), in an average sense as $T\rightarrow \infty$. We will assume the existence of the respective solutions and discuss only the convergence as $T\rightarrow \infty$. (Note that the existence of solutions is known for any dimension if $\mu$ is constant, and for $d=1$ if $\mu$ is nonconstant. For the latter case, see Section B.)

We start with the following uniform bound:

### Lemma A.1

Suppose $\inf _\Omega {m}_0(x)\ge \delta >0$. There exists a constant $C>0$ dependent on $\Vert m_0\Vert _{C^2(\bar{\Omega })}$ and $\Vert G\Vert _{C^2(\bar{\Omega })}$ independent of $T\ge 1$ such that

$$\begin{aligned} \Vert {\nabla }u^T(0,\cdot )\Vert _{L^2({\Omega })}\le C. \end{aligned}$$

### Proof of Lemma A.1

We proceed as in (Cardaliaguet et al. 2012, Lemma 1.6): multiply the first equation of (2.10) by $\partial _tm$ and the second equation by $\partial _t u$; then integrating in space proves that

$$\begin{aligned} -\int \mu \Delta u\partial _t m+\frac{1}{2}\int |{{\nabla }}u|^2\partial _tm +\int F(x,m)\partial _tm=\int \Delta (\mu m)\partial _t u-\int m\langle {{\nabla }} u,{{\nabla }}\partial _t u\rangle . \end{aligned}$$

In other words,

$$\begin{aligned} \int (-\mu \Delta u\partial _t m-\Delta (\mu m)\partial _t u)+\frac{d}{dt}\int \frac{1}{2}|{{\nabla }}u|^2m+\frac{d}{dt}\int {\tilde{F}}(x,m)=0, \end{aligned}$$

where $\tilde{F}(x,s) = \int _0^s F(x,t)\,dt$. Then, defining

$$\begin{aligned} H(t):=-\int \mu m\Delta u+\int \frac{1}{2}|{{\nabla }}u|^2m+\int \tilde{F}(x,m) \end{aligned}$$

we deduce that *H*(*t*) is constant in *t*. In particular,

A.1 $$\begin{aligned} H(0)=H(T) \end{aligned}$$

Next, using the estimate $\tilde{F}(x,m) \le (\sup _{\Omega \times [0,\infty )} F)m$ (which follows from $m\mapsto F(x,m)$ being decreasing, so that $\sup _{\Omega \times [0,\infty )}F = \sup _\Omega F(\cdot ,0)$ is finite), we deduce

$$\begin{aligned} \int _\Omega \tilde{F}(x,m)\,dx \le (\sup F) \int _\Omega m\,dx = (\sup F) \bar{m}_0 \quad \text { for any }t \in [0,T], \end{aligned}$$

where we used $\int _\Omega m(t,x)\,dx = \int _\Omega m_0\,dx= \bar{m}_0$ for all *t*. Hence,

$$\begin{aligned} H(T)\le \bar{m}_0\Vert G\Vert _{ C^2}\Vert \mu \Vert _{L^\infty }+\frac{\Vert G\Vert _{C^1}^2}{2}+ (\sup F) \bar{m}_0. \end{aligned}$$

On the other hand,

$$H(0)\ge -\int \Delta (\mu m_0) u(0,\cdot )\,dx+\frac{1}{2}\int |{{\nabla }} u(0,\cdot )|^2m_0\,dx- \sup _{x\in \Omega } |\tilde{F}(x, m_0(x))|. $$

Since $\inf _\Omega m_0\,dx \ge \delta$, We thus deduce that

A.2 $$\begin{aligned} \int |{{\nabla }} u(0,\cdot )|^2\,dx\le A + B\int \Delta (\mu m_0)u(0,\cdot )\,dx \end{aligned}$$

for two constants *A*, *B* that do not depend on *T*. Finally, observe that

A.3

where we used $\int _\Omega \Delta (\mu m_0)\,dx = 0$ due to the no-flux boundary condition for the first equality, and Poincaré’s inequality for the last inequality. Combining (A.2) and (A.3), we deduce that

$$\begin{aligned} \Vert {{\nabla }} u(0,\cdot )\Vert _{L^2}^2 \le A + BC \Vert {{\nabla }} u(0,\cdot )\Vert _{L^2}. \end{aligned}$$

This proves the boundedness of $\Vert {{\nabla }} u(0,\cdot )\Vert _{L^2}$. $\square$

Next, we recall the following special identity.

### Lemma A.2

It holds that

A.4 $$\begin{aligned}&\frac{1}{2}\iint (m^T + {\bar{m}}) |\nabla u^T - \nabla {\bar{u}}|^2\,dxdt -\iint (F(x,m^T)-F(x,{\bar{m}}))\eta \,dxdt \nonumber \\ &=\int _{{\Omega }}(v(0,\cdot )\eta (0,\cdot )-v(T,\cdot )\eta (T,\cdot ))\,dx . \end{aligned}$$

where $$v:=u^T-{\overline{u}}$$, $$\eta :=m^T-{\overline{m}}$$.

### Proof

Using the notation $v:=u^T-{\overline{u}}$, $\eta :=m^T-{\overline{m}}$, we have the system

A.5 $$\begin{aligned} {\left\{ \begin{array}{ll} -\overline{\lambda }-\partial _t v-\mu \Delta v+\frac{1}{2}(\vert {{\nabla }} u^T\vert ^2-\vert {{\nabla }} {\overline{u}}\vert ^2)=-F(x,m^T)+F(x,{\overline{m}})\,, \\ \partial _t \eta -\Delta (\mu \eta )={\nabla }\cdot (m^T {{\nabla }} u^T )-{\nabla }\cdot ({\overline{m}}{{\nabla }} {\overline{u}}) \\ v(T,\cdot )=\Psi -{\overline{u}}, \quad \text { and }\quad \eta (0,\cdot )=m_0-{\overline{m}}. \end{array}\right. } \end{aligned}$$

Next, we multiply the first equation by $\eta =m^T-{\bar{m}}$ and the second equation by $v = u^T - {\bar{u}}$, integrate by parts and subtract the result to obtain (the terms containing $m^T$ and ${\bar{m}}$ are separated in the second equality)

$$\begin{aligned}&-\iint (F(x,m^T)-F(x,{\bar{m}}))\eta \,dxdt + \int (v(T,\cdot ) \eta (0,\cdot ) - v(0,\cdot ) \eta (0,\cdot ))\,dx\nonumber \\&\quad = \iint \frac{\vert {{\nabla }} u^T\vert ^2-\vert {{\nabla }} {\overline{u}}\vert ^2}{2}(m^T-{\overline{m}})\,dtdx\\&\qquad -\iint \langle {{\nabla }} v,{{\nabla }} u^T\rangle m^T\,dtdx+\iint \langle {{\nabla }} v,{{\nabla }}{\overline{u}}\rangle {\overline{m}}\,dtdx\\&\quad = \iint \left[ \langle \nabla v, \frac{\nabla u^T + \nabla {\bar{u}}}{2}\rangle - \langle \nabla v, \nabla u^T\rangle \right] m^T \\&\qquad - \iint \left[ \langle \nabla v, \frac{\nabla u^T + \nabla {\bar{u}}}{2}\rangle - \langle \nabla v, \nabla {\bar{u}}\rangle \right] {\bar{m}}\\&\quad =\frac{1}{2} \iint \langle \nabla v, -\nabla v\rangle m^T - \frac{1}{2} \iint \langle \nabla v, \nabla v\rangle {\bar{m}} \\&\quad = -\frac{1}{2} \iint |\nabla v|^2 (m^T + {\bar{m}}). \end{aligned}$$

This proves the lemma. $\square$

### Corollary A.3

The ergodic problem (2.11) has at most one classical solution.

### Proof

For $i=1,2$, let $(\bar{\lambda }_i, {\bar{u}}_i, {\bar{m}}_i)$ be two solutions to (2.11). Similar as above, one can prove

$$\begin{aligned} \frac{1}{2}\int ( \bar{m}_2 + \bar{m}_1) |\nabla \bar{u}_2 - \nabla \bar{u}_1|^2\,dx - \int (F(x,{\bar{m}}_2) - F(x,{\bar{m}}_1))(\bar{m}_2 - \bar{m}_1) \,dx =0. \end{aligned}$$

Since $s\mapsto F(x,s)$ is strictly decreasing, it follows that

$$\begin{aligned}(\nabla \bar{u}_2,{\bar{m}}_1) \equiv (\nabla \bar{u}_1,\bar{m}_1).\end{aligned}$$

Since we normalize so that $\int _\Omega (\bar{u}_2 - \bar{u}_1) \,dx = 0$, we conclude that $\bar{u}_2 - \bar{u}_1\equiv 0$. $\square$

### Lemma A.4

Let $$v:=u^T-{\overline{u}}$$, $$\eta :=m^T-{\overline{m}}$$, then

$$\begin{aligned} \left| \int v(0,\cdot ) \eta (0,\cdot )\,dx \right| + \left| \int v(T,\cdot ) \eta (T,\cdot )\,dx \right| \le C'.\end{aligned}$$

### Proof

Using $\int _\Omega \eta (t,\cdot )\,dx = 0$ for all *t* and Poincaré’s inequality, we have

It then follows from Lemma A.1 that

$$ \left| \int v(0,\cdot )\eta (0,\cdot )\right| \le C \Vert m_0 - {\bar{m}}\Vert _{L^2(\Omega )} \Vert \nabla u(0,\cdot )-\nabla \bar{u}\Vert _{L^2(\Omega )} \le C', $$

for some constant $C'$ independent of time.

Next, we observe that $|v(T,\cdot ) \eta (T,\cdot )| \le |v(T,\cdot )| m^T(T,\cdot ) + |v(T,\cdot )| {\bar{m}}(\cdot )$. Hence,

$$\begin{aligned} \left| \int v(T,\cdot ) \eta (T,\cdot )\,dx \right|&\le C (\int m^T(T,\cdot )\,dx + \int {\bar{m}}\,dx) \le 2C {\bar{m}}_0, \end{aligned}$$

since $v(T,\cdot ) = G - {\bar{u}}$ is bounded uniformly in $L^\infty (\Omega )$. $\square$

### Proof of Proposition 2.1(a)

Using the identity (A.4) and Lemma A.4, we obtain

A.6 $$\begin{aligned} \frac{1}{2} \iint (m^T + {\bar{m}}) |\nabla v|^2\,dtdx - \iint (F(x,m^T) - F(x,{\bar{m}}))(m^T - {\bar{m}})\,dtdx \le C'. \end{aligned}$$

Using $\inf {\bar{m}} >0$ and that $s\mapsto F(x,s)$ is decreasing (thanks to (F1)), (2.14) and (2.15) follow by the change of variable $s = t/T$. $\square$

### Proof of Proposition 2.1(b)

Next, assume (F2), then it follows from (2.14) and (2.15) that

A.7 $$\begin{aligned} \Vert \nu ^T - {\bar{m}}\Vert _{L^2((0,1)\times \Omega )}+ \Vert \nabla \theta ^T - \nabla {\bar{u}}\Vert _{L^2((0,1)\times \Omega )} \le \frac{C}{T} \quad \text { as }~T\rightarrow \infty . \end{aligned}$$

This proves (2.16).

Next, we claim that

A.8 $$\begin{aligned} \Vert F(x,\nu ^T(s,x)) - F(x, {\bar{m}}(x))\Vert _{L^1([0,1]\times \Omega )} \rightarrow 0. \end{aligned}$$

Indeed, by (F2), there exists $\delta >0$ such that

$$\begin{aligned} \delta (s'-s) \le F(x,s) - F(x,s') \le \frac{1}{\delta } (s'-s) \quad \text { for }x \in \Omega ,~ 0 \le s \le s' \le 2\Vert {\bar{m}}\Vert _\infty . \end{aligned}$$

Then from (A.6) and the fact that $(F(x,s')-F(x,s))(s'-s) \le 0$, we have

$$\begin{aligned} \frac{C}{T}&\ge \iint _{[0,1]\times \Omega } \left| (F(x,\nu ^T) - F(x,{\bar{m}}))(\nu ^T - {\bar{m}})\right| \,dxds\\&\ge \Vert {\bar{m}}\Vert _\infty \iint _{\{\nu ^T \ge 2\Vert {\bar{m}}\Vert _{\infty } \}} |F(x,\nu ^T) - F(x,{\bar{m}})| \,dxdt \\&\qquad + c' \iint _{\{\nu ^T < 2\Vert {\bar{m}}\Vert _{\infty } \}} |\nu ^T - {\bar{m}}|\,dxdt. \end{aligned}$$

Therefore,

$$\begin{aligned}&\Vert F(x,\nu ^T) - F(x,{\bar{m}})\Vert _{L^1([0,1]\times \Omega )}\\&\quad \le \iint _{\{\nu ^T \ge 2\Vert {\bar{m}}\Vert _{\infty } \}} |F(x,\nu ^T) - F(x,{\bar{m}})| \,dxdt \\&\quad \quad + \iint _{\{\nu ^T< 2\Vert {\bar{m}}\Vert _{\infty } \}} |F(x,\nu ^T) - F(x,{\bar{m}})| \,dxdt\\&\quad \le \iint _{\{\nu ^T \ge 2\Vert {\bar{m}}\Vert _{\infty } \}} |F(x,\nu ^T) - F(x,{\bar{m}})| \,dxdt + \frac{1}{\delta } \iint _{\{\nu ^T <2\Vert {\bar{m}}\Vert _{\infty } \}} |\nu ^T-{\bar{m}}| \,dxdt\\&\quad \le \frac{C}{T}\left( \frac{1}{\Vert {\bar{m}}\Vert _\infty } + \frac{1}{\delta c'} \right) . \end{aligned}$$

This proves (A.8).

Next, integrate (2.11) over $\Omega$ to get

A.9

Similarly, we integrate (2.10) over $[0,t]\times \Omega$, and change variables $\theta ^T(s,x)=u^T(sT,x)$ to get

A.10

It follows from (A.7) to (A.10) that

A.11

uniformly for $s\in [0,1]$. Using Poincaré’s inequality, it follows that

A.12

The convergence of $\frac{1}{T} \theta ^T(s,x)$ to $(1-s)\bar{\lambda }$ in $L^2([0,1]\times \Omega ))$ follows by combining (A.11) and (A.12). $\square$

## Existence Results

B.1 $$\begin{aligned} {\left\{ \begin{array}{ll} \bar{\lambda }- \mu \Delta {\bar{u}} + H(x,\nabla {\bar{u}}) = V[m] & \text { in }\Omega ,\\ -\Delta (\mu {\bar{m}}) - \textrm{div} ({\bar{m}} D_p H(x,\nabla {\bar{u}})) = 0 & \text { in }\Omega ,\\ \int _\Omega {\bar{m}}\,dx = m_0,\quad \text { and }\quad \int _\Omega {\bar{u}} \,dx = 0, & \\ \partial _\nu (\mu {\bar{m}}) = 0 = \partial _\nu {\bar{u}} & \text { on }\partial \Omega . \end{array}\right. } \end{aligned}$$

We will prove the existence of classical solution $(\bar{\lambda },{\bar{u}}, {\bar{m}}) \in \mathbb {R}\times C^{2+\alpha }(\bar{\Omega }) \times W^{1,2}(\Omega )$ under the following hypotheses:

1. $\Omega$ is a bounded smooth domain in $\mathbb {R}^d$ and $\mu \in C^3(\overline{\Omega })$ satisfies $\inf _\Omega \mu >0$.
2. For each $p > d$, there exists $\alpha \in (0,1)$ such that
  B.2 $$\begin{aligned} V[m] \in C^\alpha (\overline{\Omega })\quad \text { for every }m \in W^{1,p}(\Omega ), \end{aligned}$$
  and there exists $K \in C^\alpha (\overline{\Omega })$ such that
  B.3 $$\begin{aligned} -K(x) \le V[m] \le m(x) - K(x) \text { for all }m \in W^{1,p}(\Omega ) \cap P(\Omega ). \end{aligned}$$
  Moreover, for each $k \in \mathbb {N}$, and $m_n,m \in (W^{1,p}(\Omega )\cap P(\Omega ))$
  B.4 $$\begin{aligned} \Vert m_n - m\Vert _\infty \rightarrow 0 ~\Longrightarrow ~ \Vert \min \{k,V[m_n]\} - \min \{k,V[m]\}\Vert _\infty \rightarrow 0 \end{aligned}$$
3. For some $\alpha \in (0,1)$, $H \in C^\alpha _{loc}(\overline{\Omega }\times \mathbb {R}^d)$ and for some $A_i >0$
  $$\begin{aligned} {A}_1( |p|^2- 1) \le H(x,p) \le A_2 (|p|^2 + 1) \quad \text { and }\quad |D_p H(x,p)|^2 \le A_3 +A_4H(x,p)) \end{aligned}$$
4. $$d=1$$, i.e. $$\Omega = (0,1)$$.

### Remark B.1

In application, we take $\bar{\mu }(x) = \epsilon \mu (x)$ and $H(x,p) = |p|^2.$

### Apriori estimates for the ergodic problem

#### Lemma B.2

Assume (H1) - (H3). Suppose $(\bar{\lambda }, {\bar{u}}, {\bar{m}}) \in \mathbb {R} \times C^{2+\alpha }(\bar{\Omega }) \times W^{1,p}(\Omega )$ (for some $p>d$) is a solution of (B.1), then

B.5 $$\begin{aligned} \bar{\lambda }\ge - \sup _\Omega H(\cdot , 0) -\sup _\Omega K \quad \text { and }\quad \bar{\lambda }\int _\Omega \frac{1}{\mu }\,dx + \int _\Omega \frac{H(x,\nabla {\bar{u}})}{\mu }\,dx \le \frac{m_0 }{\inf \limits _\Omega \mu },\nonumber \\ \end{aligned}$$

In particular,

B.6 $$\begin{aligned} |\bar{\lambda }| + A_1 \int _\Omega |\nabla {\bar{u}}|^2\,dx \le A_1 + \Vert H(\cdot ,0)\Vert _\infty + \Vert K\Vert _\infty + \frac{m_0 \sup \limits _\Omega \mu }{\inf \limits _\Omega \mu } \end{aligned}$$

Furthermore, there exists $C_0=C_0(m_0, \inf \limits _\Omega \mu , \sup \limits _\Omega \mu )$ such that

B.7 $$\begin{aligned} \int _\Omega \left| \log \nabla (\mu {\bar{m}})\right| ^2\,dx \le C_0. \end{aligned}$$

#### Remark B.3

If we replace $\mu$ by $\epsilon \mu$ and take $H(x,p) = |p|^2$, then we have

B.8 $$\begin{aligned} |\bar{\lambda }| + \int _\Omega |\nabla {\bar{u}}|^2\,dx \le \Vert H(\cdot ,0)\Vert _\infty + \Vert K\Vert _\infty + \frac{m_0 \sup \limits _\Omega \mu }{\inf \limits _\Omega \mu }. \end{aligned}$$

Note that $|\bar{\lambda }|$ and $\Vert \nabla {\bar{u}}\Vert _{L^2}$ are bounded uniformly in $\epsilon >0$.

#### Proof

First, we prove the lower bound of $\bar{\lambda }$ by the idea in (Lou and Ni 1999, Lemma 2.1). Let $x_0$ be the global minimum point of ${\bar{u}}$, we claim that

B.9 $$\begin{aligned} \bar{\lambda }+ H(x_0,\nabla {\bar{u}}(x_0)) \ge {\bar{m}}(x_0) - K(x_0). \end{aligned}$$

If $x_0 \in \Omega$, then (B.9) follows from classical maximum principle. Suppose $x_0 \in \partial \Omega$ and that (B.9) does not hold, then it follows by continuity that there is a neighborhood $\mathcal {O}$ of $x_0$ in $\bar{\Omega }$ such that $-\Delta {\bar{u}}>0$ in $\mathcal {O}$. By Hopf’s lemma, it follows that $\partial _\nu {\bar{u}} <0$. This is impossible since ${\bar{u}}$ satisfies the homogeneous Neumann boundary condition. Hence, (B.9) holds. It follows from (B.9) and the fact that $\nabla {\bar{u}}(x_0)=0$ (using the Neumann boundary condition again if $x_0 \in \partial \Omega$) that $\overline{\lambda }\ge -\sup _\Omega H(\cdot ,0) - K(x_0)$. This proves the lower bound in (B.5).

For the upper bound of $\bar{\lambda }$, we divide the first equation of (B.1) by $\mu (x)$ and integrate to obtain (using $K(x) \ge 0$ and the homogeneous Neumann boundary condition)

B.10 $$\begin{aligned} \int _\Omega \frac{1}{\mu }\left( \bar{\lambda }+ H(x,\nabla {\bar{u}})\right) \,dx \le \int _\Omega \frac{{\bar{m}}}{\mu }\,dx \le \frac{\int _\Omega {\bar{m}}\,dx}{\inf \mu }. \end{aligned}$$

The upper bound in (B.5) follows.

It remains to prove (B.7). To this end, we divide the second equation of (B.1) by $\mu {\bar{m}}$, and integrate by parts to get

$$\begin{aligned} \int _\Omega |\nabla \log (\mu {\bar{m}})|^2\,dx&= \int _\Omega \frac{1}{\mu {\bar{m}}} \Delta (\mu {\bar{m}})\,dx\\&= \int {\bar{m}}D_pH(x,\nabla {\bar{u}}) \cdot \nabla (\tfrac{1}{\mu {\bar{m}}})\,dx\\&= \int _\Omega \tfrac{1}{\mu } D_pH(x,\nabla {\bar{u}}) \cdot \nabla \log (\mu {\bar{m}})\,dx \\&\le \frac{1}{2} \int _\Omega |\nabla \log (\mu {\bar{m}})|^2\,dx + \frac{1}{2(\inf \mu )^2} \int _\Omega |D_pH(x,\nabla {\bar{u}})|^2 \,dx. \end{aligned}$$

Hence,

$$\begin{aligned} \int _\Omega |\nabla \log (\mu {\bar{m}})|^2\,dx \le \frac{1}{(\inf \mu )^2} \int _\Omega |D_pH(x,\nabla {\bar{u}})|^2 \,dx \le C(1 + \int _\Omega H(x,\nabla {\bar{u}})\,dx) \end{aligned}$$

where we used (H3). Combining with (B.10), we obtain (B.7) $\square$

#### Lemma B.4

Assume (H1) -(H3) and assume $\Omega = (0,1)$. Let $(\bar{\lambda }, {\bar{u}}, {\bar{m}}) \in \mathbb {R} \times C^{2+1/2}([0,1]) \times W^{1,2}([0,1])$ be a solution of (B.1), then there are constants $C_1>0$ depending on $\sup _{[0,1]} K$, $\inf _{[0,1]} \mu$ and $\Vert \mu \Vert _{C^{2+1/2}([0,1])}$ such that

B.11 $$\begin{aligned} |\bar{\lambda }| + \Vert {\bar{m}}\Vert _{W^{1,2}(\Omega )} + \Vert {\bar{u}}\Vert _{W^{2,\infty }(\bar{\Omega })} \le C_1, \end{aligned}$$

#### Proof

The bound for $|\bar{\lambda }|$ (B.11) is due to Lemma (B.2).

Next, we estimate $\Vert {\bar{m}}\Vert _\infty$.

B.12 $$\begin{aligned} |\log {\bar{m}}(x_1) - \log {\bar{m}}(x_2)| \le \int _{x_1}^{x_2} |( \log {\bar{m}})_x|\,dx \le \Vert ( \log {\bar{m}})_x\Vert _{L^2([0,1])} |x_1-x_2|^{1/2}.\nonumber \\ \end{aligned}$$

Note that $\Vert ( \log {\bar{m}})_x\Vert _{L^2([0,1])}$ is bounded, by (B.7). Thanks to (B.12), the Harnack inequality holds for *m*, i.e. there is a constant $C'$ such that

B.13 $$\begin{aligned} \sup _{[0,1]} {\bar{m}} \le C' \inf _{[0,1]}{\bar{m}}. \end{aligned}$$

Since the left hand side is bounded from above by $C'\int _\Omega {\bar{m}}= C'm_0$, we deduce that ${\bar{m}}$ is bounded uniformly.

Next, we estimate $\Vert {\bar{u}}\Vert _\infty$. To this end, let $x_0$ be the maximum point of $|\bar{u}_x|^2$, then by the first equation of (B.1),

$$\begin{aligned} A_1|\bar{u}_x(x_0)|-A_2 \le H(x_0,\bar{u}_x(x_0)) +\bar{\lambda }\le V[m]\le {\bar{m}}(x_0) - {\bar{K}}(x_0) \le C'' \end{aligned}$$

where we used hypothesis (H3) for the first inequality, $\bar{u}_{xx}(x_0) \le 0$ for the second one, and (H2) for the next to last one. Thus $\Vert \bar{u}_x\Vert _\infty$ is bounded. By multiplying the equation of ${\bar{m}}$ by $\mu {\bar{m}}$ and integrating by parts, it follows from ( Bardi and Feleqi (2016), Lemma 2.3) that

B.14 $$\begin{aligned} \Vert \mu {\bar{m}}\Vert _{W^{1,2}(\Omega )} \le C(1+\Vert \bar{u}_x\Vert _\infty ) . \end{aligned}$$

We supply the proof of (B.14) for the convenience of the reader. Indeed, for any $\varphi \in C(\bar{\Omega })$ satisfying the Neumann boundary condition, the definition of ${\bar{m}}$ as weak solution implies

$$\begin{aligned} \int _0^1 [(\mu {\bar{m}})_x + {\bar{m}} D_pH(x,(x,\bar{u}_x)) ]\varphi _x \,dx = 0. \end{aligned}$$

Hence,

$$\begin{aligned} \left| \int _0^1 (\mu {\bar{m}})_{x} \varphi _x\,dx\right|&=\left| \int _0^1{\bar{m}} D_pH(x,\bar{u}_x)\varphi _x\,dx\right| \\&\le C\int _0^1 {\bar{m}} ( 1 + |\bar{u}_x|) |\varphi _x|\,dx \le \frac{C}{\inf \mu } (1+\Vert \bar{u}_x\Vert _\infty ) \Vert \mu {\bar{m}}\Vert _2 \Vert \varphi _x\Vert _2. \end{aligned}$$

This implies that $\Vert (\mu {\bar{m}})_x\Vert _2 \le C\Vert \bar{u}_x\Vert _\infty$. Combining with (B.13), we obtain (B.14). Since $\inf \mu >0$ and $\mu \in C^2(\bar{\Omega })$, we also obtain the bound for $\Vert {\bar{m}}\Vert _{W^{1,2}([0,1])}$.

Finally, because we are in one-spatial dimension, it follows that

$$\begin{aligned} \Vert {\bar{m}}\Vert _{C^{1/2}([0,1])} \le C \Vert {\bar{m}}\Vert _{W^{1,2}([0,1])}\le C. \end{aligned}$$

We can then deduce from the first equation of (B.1) that $\Vert \bar{u}_{xx}\Vert _\infty \le C$. Combining with the bound for $\Vert {\bar{u}}\Vert _\infty$, we obtain the bound for $\Vert {\bar{u}}\Vert _{W^{2,\infty }([0,1])}$. $\square$

Next, we prove the existence of classical solution to (B.1)

#### Theorem B.5

Assume (H1)-(H3) and suppose $\Omega = (0,1)$. Then the following hold.

(i) There exists at least one solution $(\bar{\lambda },{\bar{u}}, {\bar{m}}) \in C^{2+1/2}([0,1]) \times W^{1,2}([0,1])$ to the ergodic MFG system (B.1).
(ii) The set of solutions are uniformly bounded in $\mathbb {R}\times W^{2,\infty }([0,1]) \times W^{1,2}([0,1])$.
(iii) The solution $(\bar{\lambda },{\bar{u}}, {\bar{m}})$ is unique provided the Lasry-Lions condition holds:
  $$\begin{aligned}\int _\Omega (V[m]-V[\tilde{m}])(m - \tilde{m})\,dx >0\quad \text { if }~m, \tilde{m} \in W^{1,2}([0,1])~\text { and }~m\ne \tilde{m}.\end{aligned}$$

#### Proof

The assertion (ii) is a consequence of Lemma B.4.

The assertion (iii) is well known. (Multiply the equation of $(u_1 - u_2)$ by $(m_1 - m_2)$ and vice versa.)

Next, we prove assertion (i) regarding the existence of solution $({\bar{u}}, {\bar{m}})$ to (B.1) with $V[m(\cdot )]$. We proceed by approximation. For each $k \in \mathbb {N}$, we apply the existence results of Bardi and Feleqi (2016) to the problem (B.1) with $V_k[m]= \min \{k, V[m]\}$. To this end, we verify the following two conditions (which correspond to the conditions (B.7) and (B.32) therein) holds:

$$\begin{aligned} \forall m_n,m \in (W^{1,p}(\Omega )\cap P(\Omega )), \quad \Vert m_n - m\Vert _\infty \rightarrow 0 ~\Longrightarrow ~ \Vert V_k[m_n] - V_k[m]\Vert _\infty \rightarrow 0 \end{aligned}$$

(thanks to (B.4)) and

$$\begin{aligned} \sup _{m \in (W^{1,p}(\Omega )\cap P(\Omega ))} \Vert V_k[m]\Vert _\infty <\infty . \end{aligned}$$

Hence, by (Bardi and Feleqi 2016, Theorem 2.6) (see Theorem B.6 below), there exists $(\bar{\lambda }_k, \bar{u}_k, \bar{m}_k) \in \mathbb {R} \times C^{2+1/2}([0,1]) \times W^{1,2}([0,1])$ which solves (B.1) with $V_k[m]= \min \{k,V[m]\}$.

By Lemma B.4, $\{(\bar{\lambda }_k, \bar{u}_k, \bar{m}_k)\}_{k=1}^\infty$ is bounded uniformly in $\mathbb {R}\times W^{2,\infty }\times W^{1,2}$. Passing to a subsequence, we have

$$\begin{aligned} \bar{\lambda }_k \rightarrow \bar{\lambda },\quad \bar{u}_k\rightharpoonup {\bar{u}} ~\text { in }W^{2,2}([0,1]) \quad \text { and }\quad \bar{m}_k \rightharpoonup {\bar{m}} ~\text { in }W^{1,2}([0,1]), \end{aligned}$$

and that $(\bar{\lambda }, {\bar{u}}, {\bar{m}}) \in \mathbb {R} \times W^{2,2}([0,1]) \times W^{1,2}([0,1])$ is a solution to (B.1). Finally, ${\bar{m}} \in C^{1/2}([0,1])$ by Sobolev embedding, and ${\bar{u}} \in C^{2,1/2}([0,1])$ by the first equation of (B.1). $\square$

### Existence results from Bardi et al.

For completeness, we state some existence results due to Bardi and Feleqi (2016) for the ergodic problem (B.1).

#### Theorem B.6

(Bardi and Feleqi (2016), Theorem 2.6) Suppose *V*[*m*] verifies

$$\begin{aligned} \forall m_n,m \in (W^{1,p}(\Omega )\cap P(\Omega )), \quad \Vert m_n - m\Vert _\infty \rightarrow 0 ~\Longrightarrow ~ \Vert V[m_n] - V[m]\Vert _\infty \rightarrow 0 \qquad (\textrm{B}.15) \end{aligned}$$

and

$$\begin{aligned} \qquad \qquad \qquad \qquad \sup _{m \in (W^{1,p}(\Omega )\cap P(\Omega ))} \Vert V[m]\Vert _\infty <\infty .\qquad \qquad \qquad \qquad \qquad \qquad \qquad \qquad \quad (\textrm{B}.16) \end{aligned}$$

and that *H* satisfies the natural growth condition

(C2*) For some $$\alpha \in (0,1)$$,
  $$\begin{aligned} |H(p)| \le C_1 |p|^2 + C_2 \quad \text { for all }p \in \mathbb {R}^d. \end{aligned}$$

There exist ${\bar{u}} \in C^{2,\alpha }(\Omega )$, $m \in W^{1,p}(\Omega )$, for all $1 \le p < \infty$, which solve (B.1).

## Footnotes

1 We remark that the same result holds true for the more general class of *F*(*x*, *s*) which is strictly decreasing in *s* and such that $s \mapsto F(x,s) + \lambda$ has a unique root $K(x, \lambda )$.
